# Heterogeneity in Spore Aggregation and Germination Results in Different Sized, Cooperative Microcolonies in an Aspergillus niger Culture

**DOI:** 10.1128/mbio.00870-22

**Published:** 2023-01-11

**Authors:** Jun Lyu, Martin Tegelaar, Harm Post, Juan Moran Torres, Costanza Torchia, A. F. Maarten Altelaar, Robert-Jan Bleichrodt, Hans de Cock, Luis G. Lugones, Han A. B. Wösten

**Affiliations:** a Microbiology, Utrecht University, Utrecht, the Netherlands; b Biomolecular Mass Spectrometry and Proteomics, Bijvoet Center for Biomolecular Research and Utrecht Institute for Pharmaceutical Sciences, Utrecht University, Utrecht, the Netherlands; Karlsruhe Institute of Technology (KIT)

**Keywords:** fungus, *Aspergillus niger*, secretome, colony morphology, stress survival, cell factory, conidia, heterogeneity, protein secretion, spores, stress resistance

## Abstract

The fungus Aspergillus niger is among the most abundant fungi in the world and is widely used as a cell factory for protein and metabolite production. This fungus forms asexual spores called conidia that are used for dispersal. Notably, part of the spores and germlings aggregate in an aqueous environment. The aggregated conidia/germlings give rise to large microcolonies, while the nonaggregated spores/germlings result in small microcolonies. Here, it is shown that small microcolonies release a larger variety and quantity of secreted proteins compared to large microcolonies. Yet, the secretome of large microcolonies has complementary cellulase activity with that of the small microcolonies. Also, large microcolonies are more resistant to heat and oxidative stress compared to small microcolonies, which is partly explained by the presence of nongerminated spores in the core of the large microcolonies. Together, it is proposed that heterogeneity in germination and aggregation has evolved to form a population of different sized A. niger microcolonies, thereby increasing stress survival and producing a meta-secretome more optimally suited to degrade complex substrates.

## INTRODUCTION

Aspergillus niger is among the most abundant fungi worldwide. It is a saprotrophic fungus that degrades dead organic material but it can also infect plants and immunocompromised animals and humans (1). To this end, A. niger secretes a wide variety of metabolites such as organic acids and enzymes. The organic acids reduce the pH of the environment, thus inhibiting bacterial growth, while the enzymes degrade the polymers in the substrate into breakdown products that can be taken up by the fungus to serve as nutrients. The high secretion capacity makes A. niger one of the main cell factories for industrial protein and organic acid production (2, 3).

Germination of the asexual spores of A. niger, called conidia, results in hyphae that extend at their apices and that branch subapically. The resulting colonies (also called mycelium) can reach a (sub)millimeter (microcolonies) to centimeter (macro-colonies) size when grown on a solid substrate. In liquid cultures, A. niger grows as microcolonies. Microcolonies can consist of a small network of hyphae but can also have distinct central and outer zones, in the latter case known as pellets. These different types of microcolonies often coexist in liquid cultures in the lab (4, 5) and may also coexist in nature. This variation in size is due to heterogeneous aggregation of conidia (primary aggregation) and germlings (secondary aggregation) in the culture medium (6). The aggregated conidia/germlings give rise to large microcolonies, while the nonaggregated conidia/germlings result in small microcolonies.

Primary aggregation is mediated by the hydrophobic nature of the spores due to the presence of the outer hydrophobin and melanin layers. Inactivation of the A. nidulans hydrophobin genes *dewA* and *rodA* results in reduced aggregation and, consequently, in smaller microcolonies (7). A similar effect is obtained by inactivation of the *olvA* pigmentation gene in A. niger (5). Secondary aggregation shows a linear relationship with the particle growth rate (6) and thus has the most impact on microcolony size. This aggregation is mediated by α-(1, 3)-glucan in Aspergillus fumigatus (8) and Aspergillus nidulans (9).

The impact of morphology of the mycelium on composition and amounts of proteins released into the medium is not yet clear. Morphology in bioreactors is controlled by changing growth conditions such as the amount and formulation of inoculum and medium composition (1, 10). Therefore, it is not possible to conclude whether the changes in productivity are the result of the changes in mycelium morphology, the culture conditions, or both. Recently, the relation between colony size and the expression of selected genes encoding secreted proteins was assessed by analyzing individual large and small microcolonies from the same liquid shaken culture (11). It was shown that the selected genes were only expressed in a shell at the outer part of the large microcolonies. The fact that the center of these large microcolonies is not active in expression implies that small microcolonies are more productive per gram of biomass than large microcolonies.

Here, we addressed the impact of morphology that results from partial aggregation of spores and germlings on secretome composition and total protein secretion. To this end, microcolony size was controlled by germinating spores for different periods within alginate beads, after which the beads were dissolved and the microcolonies were transferred to fresh medium to analyze the secretome. We show that small microcolonies secrete more protein and have a different secretome composition compared to large microcolonies. In fact, the secretomes of small and large microcolonies are complementary, indicating that they have a synergistic effect on polymer degrading activity. We also show that large microcolonies are more stress resistant than small microcolonies, which is partly caused by ungerminated spores in the core of the former microcolonies. Together, it is proposed that heterogeneity in spore germination and spore/germling aggregation has evolved to form a population of different sized A. niger microcolonies that performs better with respect to substrate degradation and stress survival compared to homogenous-sized microcolonies.

## RESULTS

### Impact of spore encapsulation on morphology.

**FIG 1:**
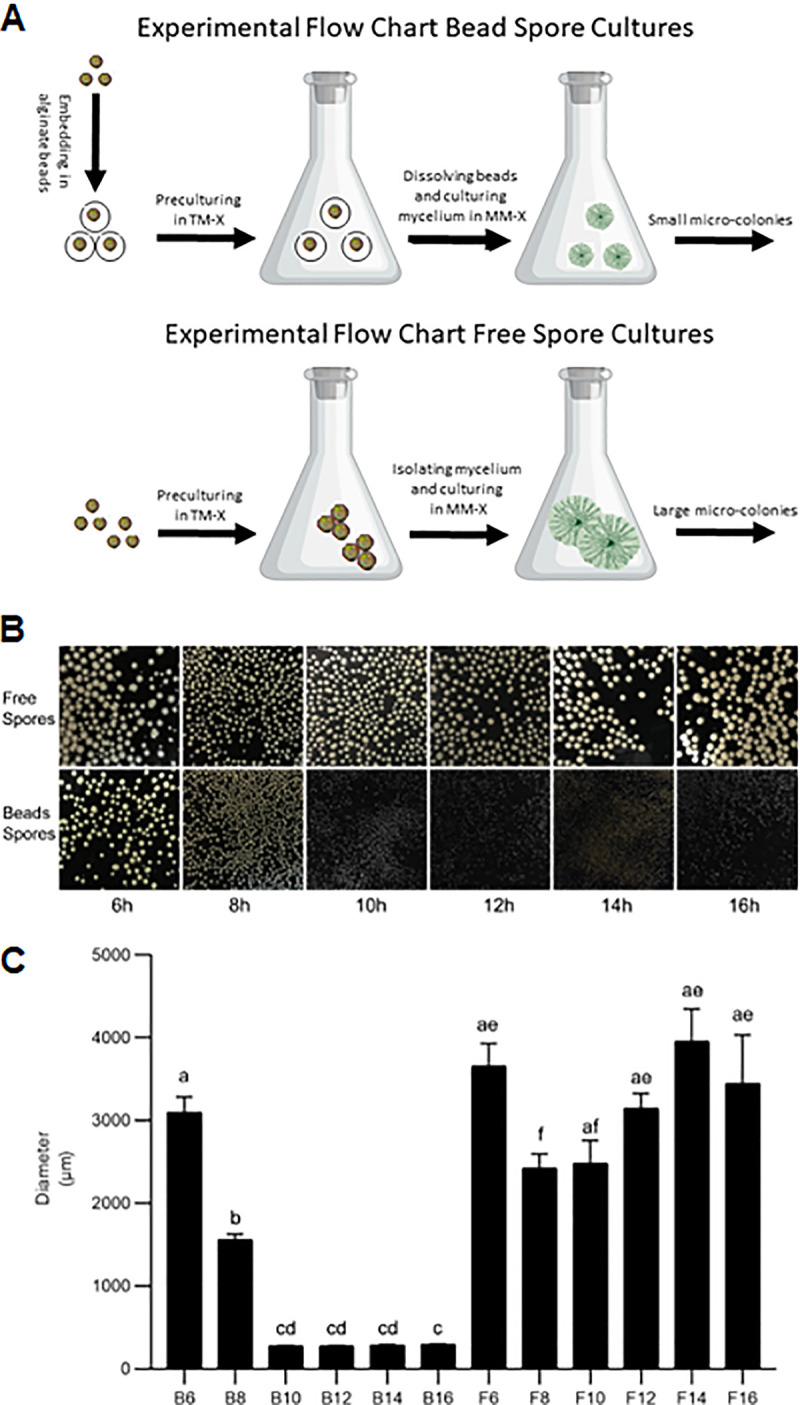
Flow chart of growing bead (B) and free (F) spore cultures (A) and the resulting morphology of the cultures after 48 h of growth (B). Beads were dissolved after 6 to 16 h of preculturing and the mycelium was transferred to MM-X for 42 to 32 h for a total culturing time of 48 h (B6-B16 cultures). Similar preculturing and culturing conditions were used for the F6-F16 cultures. (C) Microcolony diameter as calculated by image analysis. Different letters indicate statistical differences as determined by a one-way ANOVA combined with a Dunnet’s T3 *post hoc* test. Error bars indicate standard deviation.

Alginate-embedded conidia (called bead spores) were grown for 6 to 16 h in transformation medium with xylose as carbon source (TM-X) (Fig. 1A). Nonembedded spores (called free spores) served as a control. Free spores had clustered 6 h after inoculation and had started to form germlings after 8 h (Fig. S1A). Aggregation of spores and germlings increased in time. The immobilized bead spores did not aggregate and had formed germ tubes after 12 h of inoculation (Fig. S1A).

10.1128/mbio.00870-22.8 FIG S1 Morphology of spores and germlings of 6-16 h free spores (F6-16) and bead spores (B6-B16) in TM-X liquid shaken cultures (A). Bar represents 100 μm in F6-F14 and B6-B16 and 5000 μm in F16. Free spores are clustered at 6 h and had started to form germlings at 8 h. Aggregation of spores and germlings increased in time. Spores embedded in beads did not aggregate and had formed germ tubes at 12 h (indicated by arrow heads). Morphology of mycelium (B, C) and proteins released in the culture medium (D) from free spore cultures F8 and F16, to which either or not empty beads, alginate or CaCl2 had been added. Bead spore cultures B8 and B16 served as a control. The beads had been dissolved after 8 h or 16 h of preculturing in TM-X and mycelium of the B and F precultures was transferred to MM-X for 40 and 32 h, respectively, to have a total culturing time of 48 h. Statistical analysis in C was performed with a t-test or Welch’s t-test. Download FIG S1, DOCX file, 2.5 MB. Copyright © 2023 Lyu et al. 2023 Lyu et al. https://creativecommons.org/licenses/by/4.0/ This content is distributed under the terms of the Creative Commons Attribution 4.0 International license.

**FIG 2:**
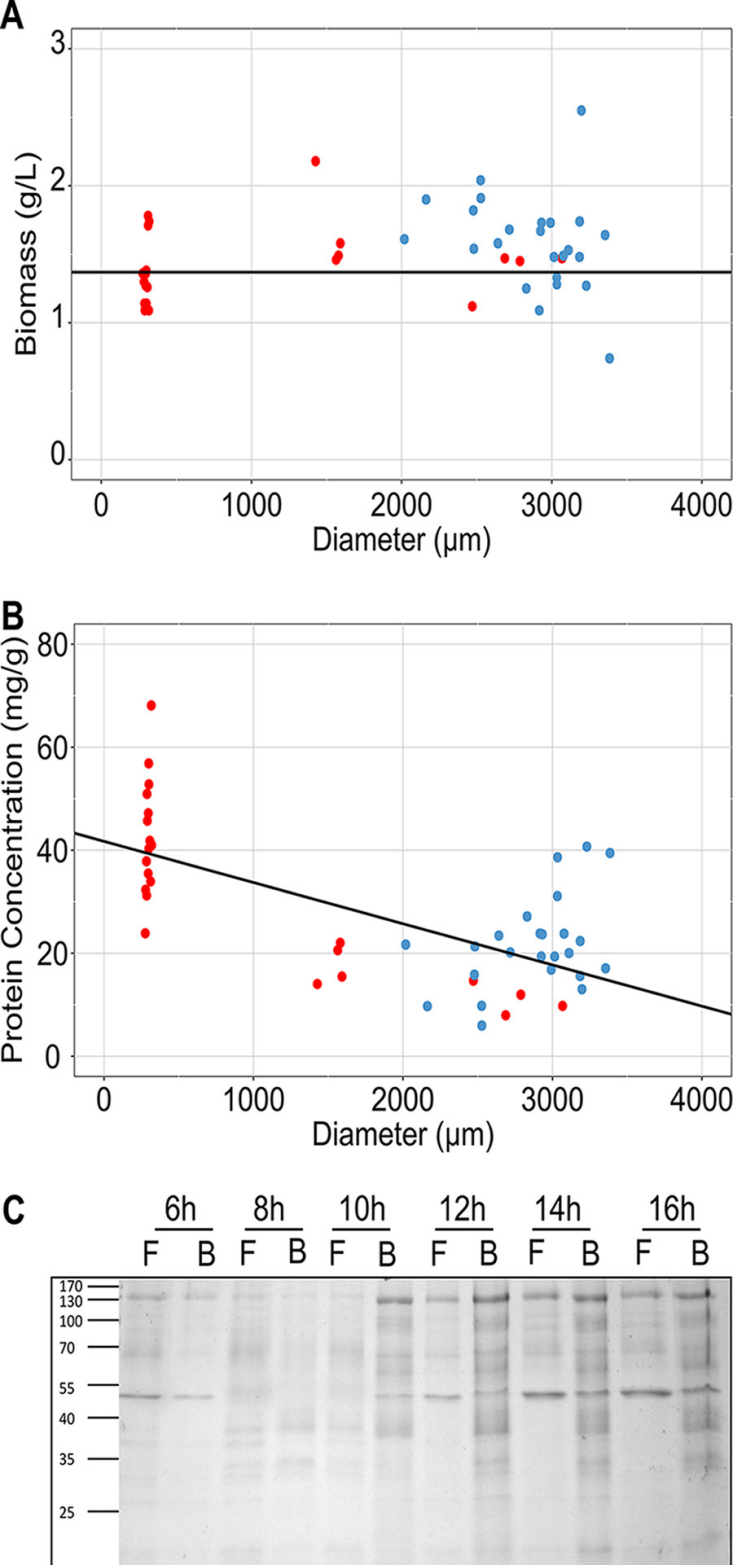
Biomass (A) and protein concentration (B) in the culture media related to the average microcolony diameter of bead spore cultures B6-B16 (red dots) and free spore cultures F6-F16 (blue dots). The relation between biomass and microcolony size and microcolony size and protein concentration in the medium were assessed by linear and quantile regression. (C) Protein profiles of the culture medium.

The beads containing the (germinated) spores were dissolved after 6 to 16 h by adding citrate buffer, after which the spores, germlings, and/or mycelium (here, collectively called mycelium) were transferred to minimal medium with xylose as carbon source (MM-X) (from now on called bead spore cultures or B-cultures) (Fig. 1A). Growth of the cultures was continued for a total culturing time of 48 h, resulting in cultures B6-B16. For instance, beads of the B6 culture were dissolved after 6 h of growth in TM-X and growth was continued for 42 h in MM-X. Similar preculturing conditions were used with nonembedded spores (from now on called free spore cultures or F-cultures) (Fig. 1A). Microcolony size of the B6-B16 and F6-F16 cultures were classified in 6 groups by statistical analysis (Fig. 1C). These 6 classes were in turn classified in 3 groups by hierarchical clustering. Group 1 consisted of mycelium of B6 cultures and the control cultures F6-F16 that had formed microcolonies of 3175 ± 610 μm (mean ± SD) (Fig. 1C). Group 2 consisted of B8 cultures that had formed intermediate sized microcolonies (1559 ± 69 μm), while group 3 consisted of cultures B10-B16 that had formed small microcolonies (285 ± 8 μm). Mean biomass of the F6-F16 and B6-B16 cultures was 1.4 ± 0.2 gr L^−1^ after 48 h of growth. Thus, biomass was not affected by microcolony size or preculturing (Fig. 2A).

In the next set of experiments, cultures were inoculated with the mycelium of 8- or 16-h-old free spore precultures that had been supplemented or not with empty beads, alginate, or CaCl_2_ to confirm that the effect on morphology was due to encapsulation in the alginate beads and not caused by chemical signals from the beads. To this end, B8 and B16 cultures were used as a control. After a total growth period of 48 h, the average diameter of the microcolonies of the B8 cultures was different compared to that of the F8 culture and the F8 culture supplemented with alginate but not different from that of the F8 cultures supplemented with CaCl_2_ or empty beads (Fig. S1BC). The latter results can be explained by the relative small size difference of the intermediate sized B8 microcolonies and the large-sized F8 cultures. In contrast, the small B16 microcolonies were smaller compared to the F16 cultures that had been supplemented or not with empty beads, alginate, or CaCl_2_. Together, the effect of beads on mycelium morphology is due to the embedding and not due to chemical induction caused by alginate and/or CaCl_2_.

### Impact of spore encapsulation on the secretome.

Total protein that had been released by the B6-B16 and F6-F16 cultures was plotted against the average microcolony size of each of the replicates (Fig. 2B). Hierarchical clustering showed that the B10-B16 cultures clustered together, while the B6-B8 and F6-F16 cultures formed the second cluster. Linear regression demonstrated that smaller colonies secrete more protein than larger microcolonies. For instance, 270 μm wide microcolonies released 2-fold more protein in the medium than 2,700 μm wide microcolonies (Fig. 2B). Also, the small sized microcolonies of the B16 cultures had released more protein than the F16 cultures containing either empty beads, alginate, or CaCl_2_, or containing no additives (Fig. S1D). This effect was less pronounced when medium of B8 cultures was compared to F8 cultures containing no additives or empty beads, alginate or CaCl_2_. These results are explained by the fact that the variation in microcolony size between the F8 and B8 cultures is less distinct compared to that of the B16 and F16 cultures. Together, the effect of embedding of spores on protein release is due to its effect on morphology and not due to chemical induction caused by alginate and/or CaCl_2_.

**FIG 3:**
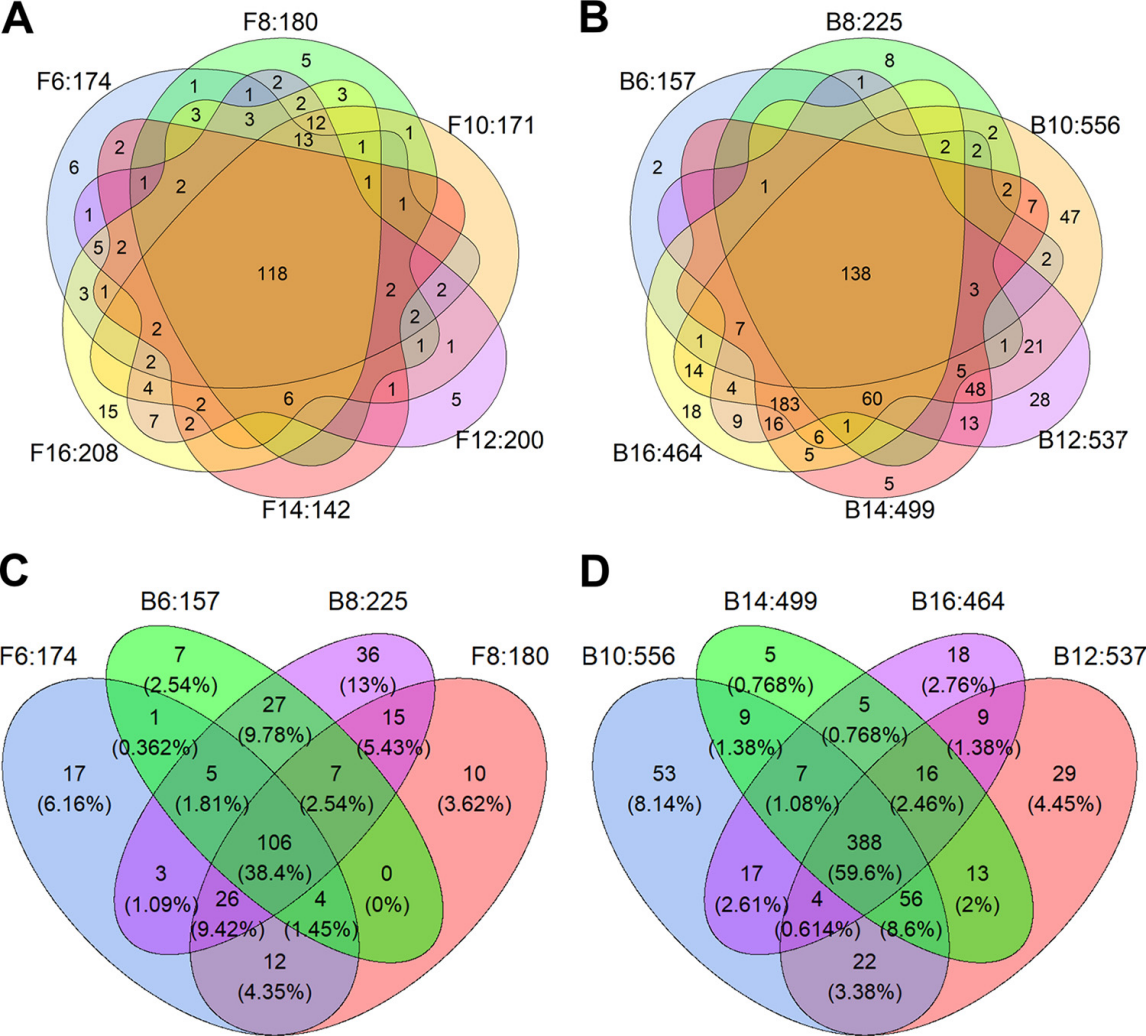
Venn diagram of the number of proteins from free spore (F6-F16) cultures (A, C) and bead spore (B6-B16) cultures (B, C, D). The beads had been dissolved just before transfer to MM-X and growth was prolonged up to a total culturing time of 48 h. The number following the F6-F16 and the B6-B16 cultures indicate the total number of proteins identified in the culture medium.

Secretomes of the F6-F16 and B6-B16 cultures (Fig. 2C) were analyzed by mass spectrometry (Table S2). A set of 93 proteins was shared between all these cultures (Fig. S2A; Table S3A). A total number of 84 proteins within this set was predicted to have a signal sequence for secretion. The F6-F16 cultures (that all formed large microcolonies) had secreted 142 to 208 proteins in the culture medium (Table 1). A set of 118 proteins was shared between these cultures, while 0 to 15 proteins were unique for each of the cultures (Fig. 3A). In contrast, the B6-B16 cultures had secreted 157 to 556 proteins (Table 1). A set of 138 proteins was shared between these cultures, while 2 to 47 proteins were unique to each of these conditions (Fig. 3B). The B6 and B8 cultures (forming large and intermediate sized microcolonies) showed the lowest number of proteins of the B-cultures (i.e., 157 and 225 proteins, respectively) similar to those of the F6-F16 cultures (forming large microcolonies) (Table 1). A set of 106 proteins was shared between the F6, F8, B6, and B8 cultures (Fig. 3C). On the other hand, the B10-B16 cultures (forming small microcolonies) shared 388 proteins in their secretome (Fig. 3D). Together, secretome diversity in cultures is lower when the pellets are larger.

**TABLE 1:** Proteins with and without a predicted signal sequence for secretion (SignalP) released into the medium of free spore (F) and bead spore (B) cultures^*a*^

Sample	SignalP	NonSignalP	Total
F6	155	19	174
B6	126	31	157
F8	158	22	180
B8	173	52	225
F10	149	22	171
B10	258	298	556
F12	170	30	200
B12	257	280	537
F14	124	18	142
B14	250	249	499
F16	179	29	208
B16	225	239	464

a Number following F and B indicates time of transfer of the precultures. Total culturing time was 48 h.

10.1128/mbio.00870-22.2 TABLE S2 Quantity (average and standard deviation) of proteins from free spore (F) and bead spore (B) cultures. Numbers following F and B indicate the time of preculturing. Download Table S2, XLSX file, 0.1 MB. Copyright © 2023 Lyu et al. 2023 Lyu et al. https://creativecommons.org/licenses/by/4.0/ This content is distributed under the terms of the Creative Commons Attribution 4.0 International license.

10.1128/mbio.00870-22.3 TABLE S3 Proteins shared in the media of free spore cultures F6-F16 and bead spore cultures B6-B16 (A) and proteins found in free spore cultures F6-16 not found in bead cultures B6-B16 (B). Download Table S3, XLSX file, 0.02 MB. Copyright © 2023 Lyu et al. 2023 Lyu et al. https://creativecommons.org/licenses/by/4.0/ This content is distributed under the terms of the Creative Commons Attribution 4.0 International license.

10.1128/mbio.00870-22.9 FIG S2 (A) Flower plot of proteins from free spore (F) and bead spore (B) cultures. The beads had been dissolved after 6-16 h of preculturing (indicated by the numbers following B) and culturing was extended for42 to 32 h after transfer of the mycelium to have a total culturing time of 48 h. Total number of proteins not being part of the core are indicated in the leaves of the flower plot. (B, C) Pairwise comparison of number (B) and quantity (C) of proteins from bead spore B6 cultures with B8, B10, B12, B14 and B16 cultures. Proteins were analyzed after a total culturing time of 48 h. The number following B indicates the time of preculturing in hours. Red and blue dots indicate proteins that were ≥ 2-fold up- or down-regulated in B6 cultures compared to the other B cultures. Download FIG S2, DOCX file, 2.0 MB. Copyright © 2023 Lyu et al. 2023 Lyu et al. https://creativecommons.org/licenses/by/4.0/ This content is distributed under the terms of the Creative Commons Attribution 4.0 International license.

**FIG 4:**
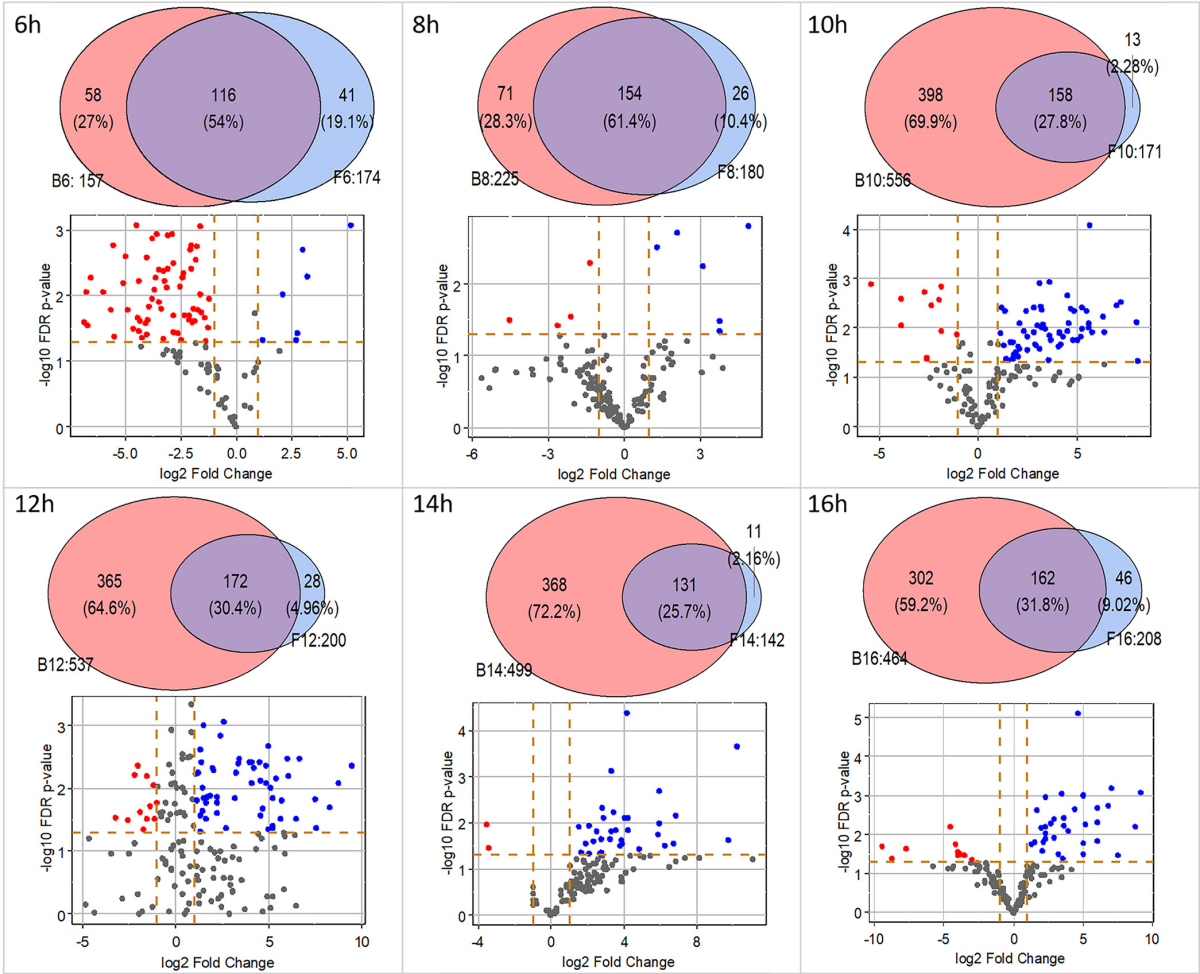
Pairwise comparison of number (top) and quantity (bottom) of proteins from liquid shaken free spore (F6-F16) cultures and bead spore (B6-B16) cultures. Proteins were analyzed after a total culturing time of 48 h. Numbers following F and B indicate the total number of proteins that had been identified in the medium. Red and blue dots indicate proteins that were ≥ 2-fold down- or upregulated in B cultures compared to F cultures, respectively.

Secretomes of the F and B cultures that had been transferred at the same time to fresh medium were pairwise compared (Fig. 4; Table S4). The number of proteins in the secretomes of the F6 and B6 cultures (both forming large microcolonies) was similar with 58 and 41 unique proteins in each of the cultures, respectively. From *t* = 8 h onwards the bead spore cultures secreted a higher variety of proteins (Fig. 4). The secretomes of the F8 cultures (forming large microcolonies) and B8 cultures (forming intermediate sized microcolonies) had released 26 and 71 unique proteins, respectively. Notably, the B10-B16 cultures (forming small microcolonies) had released > 300 unique proteins, while 11 to 46 unique proteins were identified in the media of the F10-F16 cultures (forming large microcolonies). The number of proteins that were shared between the F and B cultures of the same age was relatively constant with 116 to 172 proteins (Fig. 4).

10.1128/mbio.00870-22.4 TABLE S4 Fold changes of proteins found in B cultures compared to F cultures that had been transferred to MM-X at the same time point. Download Table S4, XLSX file, 0.05 MB. Copyright © 2023 Lyu et al. 2023 Lyu et al. https://creativecommons.org/licenses/by/4.0/ This content is distributed under the terms of the Creative Commons Attribution 4.0 International license.

Apart from proteins that were uniquely present in one of the cultures in the pairwise comparison, there were also proteins that had been released in both cultures but at different levels. A set of 7 and 65 proteins was >2-fold up- and downregulated, respectively, in the medium of B6 compared to the F6 cultures (Fig. 4; Table S4). Less than 10 proteins were up- or downregulated in the case of the B8 and F8 cultures. In contrast, a high number of proteins (≥33) were upregulated in the medium of the B10-B16 cultures compared to their respective F cultures, while only 2 to 12 proteins were downregulated. Similarly, 82 to 135 proteins were upregulated in the B8-B16 cultures (forming intermediate or small microcolonies) compared to the B6 cultures (forming large microcolonies) (Fig. S2BC). Conversely, only 0 to 3 proteins were upregulated in the B6 cultures compared to the other B cultures. Together, small microcolonies do not only produce a higher variety of unique proteins; they also produce a higher quantity of a set of proteins that are also released in the medium of large microcolonies.

### Type of proteins found in the culture medium.

Proteins may be released in the culture medium by classical secretion (presence of a signal peptide), by nonclassical secretion (absence of a signal peptide), as well as by lysis. The presence of signal peptides of the proteins in the secretome was determined to assess the potential impact of nonclassical secretion and lysis. A total of 18 to 30 proteins out of the 142 to 208 proteins in the secretomes of the F6-F16 cultures (forming large microcolonies) did not contain a predicted signal sequence for secretion (Table 1).

Protein family (Pfam) analysis (Table 2, Table S5) showed that cultures with small microcolonies (B10-B16) have more Pfam families in their secretomes compared to cultures with intermediate or large microcolonies (B6-B8 and F6-F16) (Text S1A). Next, we assessed the presence of unique proteins in the secretomes of the F and B cultures. A total of 21 and 3 unique proteins with and without a signal sequence were found in the media of F6-F16 cultures, respectively, compared to the B6-B16 cultures (Table S3B). Among the 21 unique proteins with signal sequence were CAZymes of the GH16, GH18, and GH61 families as well as a CBH cellulase (exocellobiohydrolase II; GH6) (12) that was formed by F16 cultures. A total number of 109 and 333 unique proteins with and without signal sequence, respectively, was found in the B6-B16 cultures (forming large [B6], intermediate [B8] and small microcolonies [B10-B16]) that were absent in the F-cultures, including proteins of the CAZyme families GH1, GH11, GH20, GH26, GH35, GH47, GH71, GH76, GH88, and GH92, as well as the cellulases EglB and AgsC (GH5) (12) (Table S6A). The number of unique proteins in the B10-B16 cultures was 85 and 304 with and without signal sequence, respectively (Table S6B).The two B-specific cellulases EglB and AgsC were produced by the B10-B14 cultures forming small microcolonies. Thus, small and large microcolonies secrete different cellulases into the culture medium (EglB and AgsC versus CBH).

**TABLE 2:** Number of proteins with a Pfam domain identified in free spore (F) and bead spore (B) cultures^*a*^

		F 6	F 8	F 10	F 12	F 14	F 16	B 6	B 8	B 10	B 12	B 14	B 16
Proteins with signal sequence for secretion													
PF17678	GH family 92 N-terminal domain	0	0	0	0	0	0	0	0	4	4	4	4
PF12296	Hydrophobic surface binding protein A	0	0	0	0	0	0	0	3	0	0	0	0
PF10342	Ser-Thr-rich glycosyl-phosphatidyl-inositol-anchored membrane family	3	3	3	4	3	3	4	4	5	5	5	5
PF09286	Pro-kumamolisin, activation domain	5	5	5	5	5	5	5	5	5	5	5	4
PF09260	Domain of unknown function (DUF1966)	3	3	3	3	0	3	0	4	4	4	4	4
PF08386	TAP-like protein	0	0	0	0	0	0	0	0	3	3	3	3
PF07983	X8 domain	4	0	0	0	0	3	0	0	3	3	3	0
PF07971	GH family 92	0	0	0	0	0	0	0	0	4	4	4	4
PF07732	Multicopper oxidase	0	0	0	0	0	0	0	0	0	4	4	4
PF07731	Multicopper oxidase	0	0	0	0	0	0	0	0	0	4	4	4
PF04616	GH family 43	4	5	4	5	3	3	3	5	6	6	6	5
PF03663	GH family 76	0	0	0	0	0	0	0	0	0	5	4	0
PF03443	GH family 61	0	4	0	0	0	0	0	3	0	0	3	3
PF03198	Glucanosyltransferase	6	4	4	4	4	5	5	3	6	6	6	5
PF01764	Lipase (class 3)	4	4	3	4	0	3	0	4	4	4	4	0
PF01735	Lysophospholipase catalytic domain	0	3	3	3	3	3	0	0	0	0	0	0
PF01476	LysM domain	0	0	0	0	0	3	0	0	0	0	0	0
PF00734	Fungal cellulose binding domain	0	4	0	0	0	3	0	4	4	0	4	4
PF00450	Serine carboxypeptidase	3	3	0	4	0	4	0	0	7	7	7	6
PF00394	Multicopper oxidase	0	0	0	0	0	0	0	0	0	4	4	4
PF00328	Histidine phosphatase superfamily (branch 2)	0	0	0	0	0	0	0	0	0	4	0	0
PF00150	Cellulase (GH family 5)	5	5	4	5	4	5	3	5	7	7	7	4
PF00085	Thioredoxin	0	0	0	0	0	0	0	0	4	4	0	0
PF00082	Subtilase family	3	4	4	4	3	4	3	4	4	4	4	4
Proteins without signal sequence for secretion													
PF14543	Xylanase inhibitor N-terminal	3	0	3	4	4	5	0	0	5	6	6	4
PF14310	Fibronectin type III-like domain	0	0	0	0	0	0	0	0	5	5	0	0
PF13472	GDSL-like Lipase/Acylhydrolase family	0	0	0	0	0	0	0	0	0	3	3	0
PF11976	Ubiquitin-2 like Rad60 SUMO-like	0	0	0	0	0	0	0	3	0	3	3	3
PF10584	Proteasome subunit A N-terminal signature	0	0	0	0	0	0	0	0	0	6	0	4
PF08031	Berberine and berberine like	6	5	4	4	0	6	4	7	8	7	0	0
PF07690	Major Facilitator Superfamily	0	0	0	0	0	0	0	0	0	0	1	1
PF06723	MreB/Mbl protein	0	0	0	0	0	0	0	0	4	4	4	3
PF04389	Peptidase family M28	0	0	0	0	0	0	0	0	0	4	0	0
PF04185	Phosphoesterase family	0	0	0	0	0	0	0	3	5	4	5	5
PF01915	GH family 3 C-terminal domain	0	0	0	0	0	0	0	0	5	5	0	0
PF01565	FAD binding domain	9	7	6	7	5	9	6	10	12	10	9	0
PF01328	Peroxidase, family 2	0	0	0	0	0	0	0	0	0	3	0	0
PF01055	GH family 31	0	0	0	0	0	0	3	3	0	3	3	3
PF00933	GH family 3 N-terminal domain	0	0	0	0	0	0	0	0	5	5	0	0
PF00722	GH family 16	5	5	5	5	4	5	4	5	6	6	6	6
PF00657	GDSL-like Lipase/Acylhydrolase	3	0	0	3	0	3	3	0	4	4	5	0
PF00557	Metallopeptidase family M24	0	0	0	0	0	0	0	0	0	0	0	4
PF00326	Prolyl oligopeptidase family	0	0	0	0	0	0	0	0	7	7	7	7
PF00240	Ubiquitin family	0	0	0	0	0	0	0	3	0	0	0	0
PF00227	Proteasome subunit	0	0	0	0	0	0	0	0	0	8	0	6
PF00173	Cytochrome b5-like Heme/Steroid binding domain	0	0	0	0	0	0	0	0	6	6	6	6
PF00128	Alpha amylase, catalytic domain	0	0	0	0	0	0	0	4	5	6	5	5
PF00083	Sugar (and other) transporter	0	0	0	0	0	0	0	0	0	0	1	1
PF00076	RNA recognition motif. (a.k.a. RRM, RBD, or RNP domain)	0	0	0	0	0	0	0	0	10	0	0	0
PF00026	Eukaryotic aspartyl protease	5	4	6	7	6	9	4	4	8	10	10	6
PF00012	Hsp70 protein	0	0	0	0	0	0	0	0	4	4	4	0

a Numbers following F and B indicate the time of preculturing in hours. Total culturing time was 48 h.

10.1128/mbio.00870-22.5 TABLE S5 Proteins with (A) and without (B) signal sequences for secretion identified in liquid shaken cultures that had been inoculated with mycelium of free (F) or bead (B) spore precultures. Download Table S5, XLSX file, 0.02 MB. Copyright © 2023 Lyu et al. 2023 Lyu et al. https://creativecommons.org/licenses/by/4.0/ This content is distributed under the terms of the Creative Commons Attribution 4.0 International license.

10.1128/mbio.00870-22.6 TABLE S6 Proteins found in bead spore cultures B6-B16 not found in free cultures F6-F16 (A) and proteins found in cultures of small microcolonies (B10-B16) not found in cultures of large microcolonies (F6-16, B6-8) (B). Total time of preculturing plus culturing was 48 h. Download Table S6, XLSX file, 0.05 MB. Copyright © 2023 Lyu et al. 2023 Lyu et al. https://creativecommons.org/licenses/by/4.0/ This content is distributed under the terms of the Creative Commons Attribution 4.0 International license.

10.1128/mbio.00870-22.10 TEXT S1 (A) Pfam analysis of F6-F16 and B6-B16 cultures. Data show that cultures with small microcolonies have more Pfam families in their secretomes compared to cultures with intermediate or large microcolonies. (B) Cellular proteomics of large (F12) and small (B12) microcolonies. A total of 15 and 25 were up- and down-regulated in small compared to large microcolonies after 6, 12, and 24 h of transfer to MM-X. The carbonic acid anhydrase protein CaaA and the transcription factor ZtfA were among the differentially expressed proteins. Inactivation of their genes did not impact their phenotype. (C) The secretome of A. niger of the different cultures was compared to the Aspertome database (https://cb.imsc.res.in/aspertome/home). This database suggests that 31 proteins are secreted by the nonclassical pathway, of which 23 without a signal sequence for secretion. Of the 31 proteins, 24 were only found in the bead spore cultures, while 13 were only present in cultures with small microcolonies (i.e., ≥10 h bead spore cultures). Download Text S1, DOCX file, 0.7 MB. Copyright © 2023 Lyu et al. 2023 Lyu et al. https://creativecommons.org/licenses/by/4.0/ This content is distributed under the terms of the Creative Commons Attribution 4.0 International license.

### Secretion of proteins encoded by XlnR regulated genes.

XlnR is a transcription factor that is activated by xylose and that regulates xylanolytic and cellulolytic encoding genes (13, 14). Depending on the carbon source, 38 genes have been found to be regulated solely by XlnR (13). B6-B16 cultures had released 14 to 23 out of the 38 XlnR regulated proteins (Table 3). The B10-B16 samples forming small microcolonies had released 19 to 23 of these proteins, while the B6 and B8 samples (forming large and intermediate microcolonies) had only released 14 to 16 of these proteins, which was similar to that of the F6-F16 cultures (forming large microcolonies) that had released 15 to 18 xylanolytic proteins. The XlnR-regulated proteins that were exclusively found in the media of small microcolonies were XarB (acting on xylan/pectin), EglB (acting on cellulose), XynB (acting on xylan), AgsC (acting on cellulose), AxlA (acting on xylan), and FaeA (acting on xylan and pectin), while the cellulase cellobiohydrolase CBH was exclusively found in the media of large microcolonies.

**TABLE 3:** Presence (X) or absence (-) of XlnR regulated proteins in free spore (F) and bead spore (B) cultures^*a*^

XlnR	Gene	GH family	Predict function	F6	F8	F10	F12	F14	F16	B6	B8	B10	B12	B14	B16
ATCC64974_109010	BGL/bglA	GH 3	Cellulose	X	X	X	X	X	X	X	X	X	X	X	X
ATCC64974_15350	BXL/xlnD	GH 3	Xylan, pectin	X	X	X	X	X	X	X	X	X	X	X	X
ATCC64974_83150	XLN/xynA	GH 10	Xylan	X	X	X	X	X	X	X	X	X	X	X	X
ATCC64974_2380	XG-EGL	GH 12	Cellulose	X	X	X	X	X	X	X	X	X	X	X	X
ATCC64974_1620	AGL	GH 27	Xyloglucan, xylan	X	X	X	X	X	X	X	X	X	X	X	X
ATCC64974_53780	AGL/aglB	GH 27	Xyloglucan, xylan	X	X	X	X	X	X	X	X	X	X	X	X
ATCC64974_13640	LAC/lacA	GH 35	Xyloglucan, xylan, pectin	X	X	X	X	X	X	X	X	X	X	X	X
ATCC64974_104980	ABF/abfC	GH 51	Xyloglucan, xylan, pectin	X	X	X	X	X	X	X	X	X	X	X	X
ATCC64974_30860	ABF/abfB	GH 54	ABF/abfB	X	X	X	X	X	X	X	X	X	X	X	X
ATCC64974_83160	AXH/axhA	GH 62	Xylan	X	X	X	X	X	X	X	X	X	X	X	X
ATCC64974_4680	AGU/aguA	GH 67	Xylan	X	X	X	X	X	X	X	X	X	X	X	X
ATCC64974_21600	XG-EGL/eglC	GH 74	Cellulose	X	X	X	X	X	X	X	X	X	X	X	X
ATCC64974_37230	AXE/axeA	CE 1	Xylan	X	X	X	X	X	X	X	X	X	X	X	X
ATCC64974_56580	Unknown	GH 3	Unknown	X	X	X	X	X	X	X	X	X	X	X	X
ATCC64974_40800	CBH	GH 6	Cellulose	X	X	X	X	-	-	-	X	X	X	X	X
ATCC64974_75710	PME/pmeA	CE 8	Pectin	X	X	X	X	X	X	-	-	X	X	X	X
ATCC64974_64540	BGL	GH 3	Cellulose	X	-	-	-	-	X	-	X	X	X	X	X
ATCC64974_87130	BGL	GH 3	Cellulose	-	-	-	-	-	X	-	-	X	X	X	X
ATCC64974_64370	BXL-BF/xarB	GH 3	Xylan, pectin	-	-	-	-	-	-	-	-	-	X	X	-
ATCC64974_49560	EGL/eglB	GH 5	Cellulose	-	-	-	-	-	-	-	-	X	X	X	-
ATCC64974_104930	CBH	GH 6	Cellulose	-	-	-	-	-	X	-	-	-	-	-	-
ATCC64974_22790	XLN/xynB	GH 11	Xylan	-	-	-	-	-	-	-	-	-	-	-	X
ATCC64974_40950	AGS/agsC	GH 13	Cellulose	-	-	-	-	-	-	-	-	X	X	-	-
ATCC64974_10330	BXL/axlA	GH 31	Xylan	-	-	-	-	-	-	-	-	X	X	X	-
ATCC64974_41050	FAE	CE 1	Xylan, pectin	-	-	-	-	-	-	-	-	-	X	X	-

a Numbers following F and B indicate the time of preculturing in hours. In all cases, total cultivation time was 48 h.

### A set of proteins with a signal sequence identified for the first time within the A. niger secretome.

Within the set of proteins identified in the F6-F16 and B6-B16 cultures, 94 proteins without signal peptide (data not shown) and 22 proteins with signal peptide had not been reported before to be part of the A. niger secretome (Table 4). These 22 proteins were identified in B cultures, while 7 of them were also identified in F cultures. Out of the 22 proteins, 6 were found in all B6-B16 cultures, while 13 were only identified in the B10-B16 cultures that formed small colonies (Table 4). The glycopeptidase PngN, an extracellular carbonic anhydrase and lactonase LctB were among these 22 proteins. The latter is involved in the conversion of glucose into gluconic acid, thereby releasing H_2_O_2._

**TABLE 4:** Proteins with a predicted signal sequence that had not been reported before to be part of the A. niger secretome^*a*^

ProteinID	Annotation	Location	Samples
ATCC64974_105570	ubiquitin-protein transferase activity (IMP)	Cytoplasm	B10-B16
ATCC64974_10480	PF10342 Ser-Thr-rich glycosyl-phosphatidyl-inositol-anchored membrane family	Integral component of membrane	F6-F16 and B6-B16
ATCC64974_105820	PF05345 Putative Ig domain	Integral component of membrane	B12-B16
ATCC64974_49750	PF00005 ABC transporter	Integral component of membrane	B12-B16
ATCC64974_90920	PF03176 MMPL family	Integral component of membrane	B10-B14
ATCC64974_10620	PF00264 Central domain of tyrosinase	Classical secretion pathway: Extracellular	B10-B16
ATCC64974_18030	PF10282 Lactonase, 7-bladed beta-propeller	Classical secretion pathway: Extracellular	B10-B16
ATCC64974_18290	PF16334 Domain of unknown function (DUF4964)	Classical secretion pathway: Extracellular	B6, B10, B12, B16
ATCC64974_25530	PF00194 Eukaryotic-type carbonic anhydrase	Classical secretion pathway: Extracellular	B10-B16
ATCC64974_42730	PF00561 alpha/beta hydrolase fold	Classical secretion pathway: Extracellular	F16, B10-B16
ATCC64974_60000	PF00857 Isochorismatase family	Classical secretion pathway: Extracellular	B10-B12
ATCC64974_74560	C3HC4 type (RING finger) Zinc finger family	Classical secretion pathway: Extracellular	F8-F12, F16, B8-B10, B14-B16
ATCC64974_75850	PF11578 Protein of unknown function (DUF3237)	Classical secretion pathway: Extracellular	B10-B14
ATCC64974_75940	PF00884 Sulfatase	Classical secretion pathway: Extracellular	B12
ATCC64974_78580	PF00328 Histidine phosphatase superfamily (branch 2)	Classical secretion pathway: Extracellular	B12
ATCC64974_84710	PF12222 Peptide N-acetyl-beta-D-glucosaminyl asparaginase amidase A	Classical secretion pathway: Extracellular	B10-B16
ATCC64974_103600	Uncharacterized protein	Unknown	F6-F12, F16, B6-B16
ATCC64974_104760	Uncharacterized protein	Unknown	F16, B10-B16
ATCC64974_18290	PF16334 Domain of unknown function (DUF4964)	Unknown	B6, B10-B16
ATCC64974_53750	PF05630 Necrosis inducing protein (NPP1)	Unknown	F8, B8-B16
ATCC64974_67640	PF10342 Ser-Thr-rich glycosyl-phosphatidyl-inositol-anchored membrane family	Unknown	B10-B16
ATCC64974_89110	PF10342 Ser-Thr-rich glycosyl-phosphatidyl-inositol-anchored membrane family	Unknown	F12, B6-B16

a Letters in the samples column refer to free spore (F) and bead spore (B) cultures. Numbers following F and B refer to the time of preculturing in hours. In all cases, total cultivation time was 48 h.

### Gene expression in microcolonies of different size.

**FIG 5:**
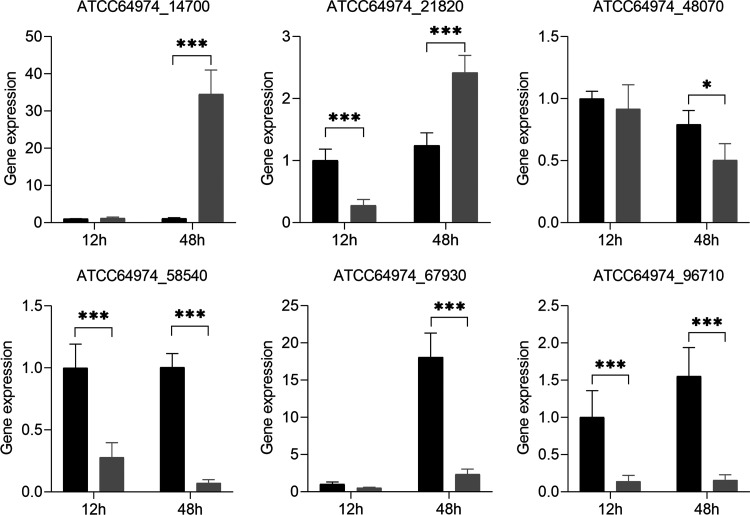
qPCR of genes ATCC64974_14700, ATCC64974_21820 and ATCC64974_48070 that encode proteins that showed higher quantities in small microcolonies (B10-B16) and of genes ATCC64974_58540, ATCC64974_96710 and ATCC64974_67930 that encode proteins that showed higher levels in large microcolonies as assessed by proteomics (F6-F16, B6-B8). Relative gene expression in free spore cultures F12 (black shaded) and bead spore cultures B12 (gray shaded) after 12 h (upon transfer to fresh medium) and 48 h (at the end of culturing). Gene expression in F12 cultures after 12 h was set at 1. *, *P* < 0.05; **, *P* < 0.01; and ***, *P* < 0.001 indicate statistical significance. Gene expression was analyzed by *t* test. Error bars indicate standard deviations.

Expression of 6 genes encoding proteins with a signal sequence for secretion was assessed in microcolonies to confirm differential protein secretion in microcolonies of different size. To this end, qPCR was used to quantify expression of genes ATCC64974_58540, ATCC64974_96710, and ATCC64974_67930 that encode proteins that showed higher levels in large microcolonies (F6-F16, B6-B8) as well as genes ATCC64974_14700, ATCC64974_21820, and ATCC64974_48070 that encode proteins that showed higher quantities in small microcolonies (B10-B16) (Table S7). RNA was extracted from F12 and B12 cultures after 12 h (at the moment that the culture was transferred to MM-X) and 48 h (at the end of culturing). The expression levels of these genes were normalized to the expression of the actin gene (Fig. 5). Expression of gene ATCC64974_48070 after 48 h was not in line with the proteomics data. In contrast, expression levels of genes ATCC64974_14700 and ATCC64974_21820 were 31.4 and 2-fold higher, respectively, after 48 h in the small compared to the large microcolonies. These differences were not yet observed upon transfer of the mycelium of the precultures to MM-X medium. Genes ATCC64974_58540, ATCC64974_67930, and ATCC64974_96170 also showed the expected gene expression after 48 h of culturing with a 7.7 to 13.9 times higher expression level in large compared to small pellets. Similar results were observed after 12 h with 1.9 to 7.1-fold differences in gene expression, although the 1.9-fold difference in gene expression of ATCC64974_67930 was not statistically significant. Together, 5 out 6 selected genes showed a similar expression level compared to protein abundance in the culture medium after 48 h of culturing.

10.1128/mbio.00870-22.7 TABLE S7 Proteins that are only highly expressed in cultures with large (B6-8,F6-16) or small microcolonies (B10-B16). Download Table S7, XLSX file, 0.01 MB. Copyright © 2023 Lyu et al. 2023 Lyu et al. https://creativecommons.org/licenses/by/4.0/ This content is distributed under the terms of the Creative Commons Attribution 4.0 International license.

### Cellulase activity in the medium.

**FIG 6:**
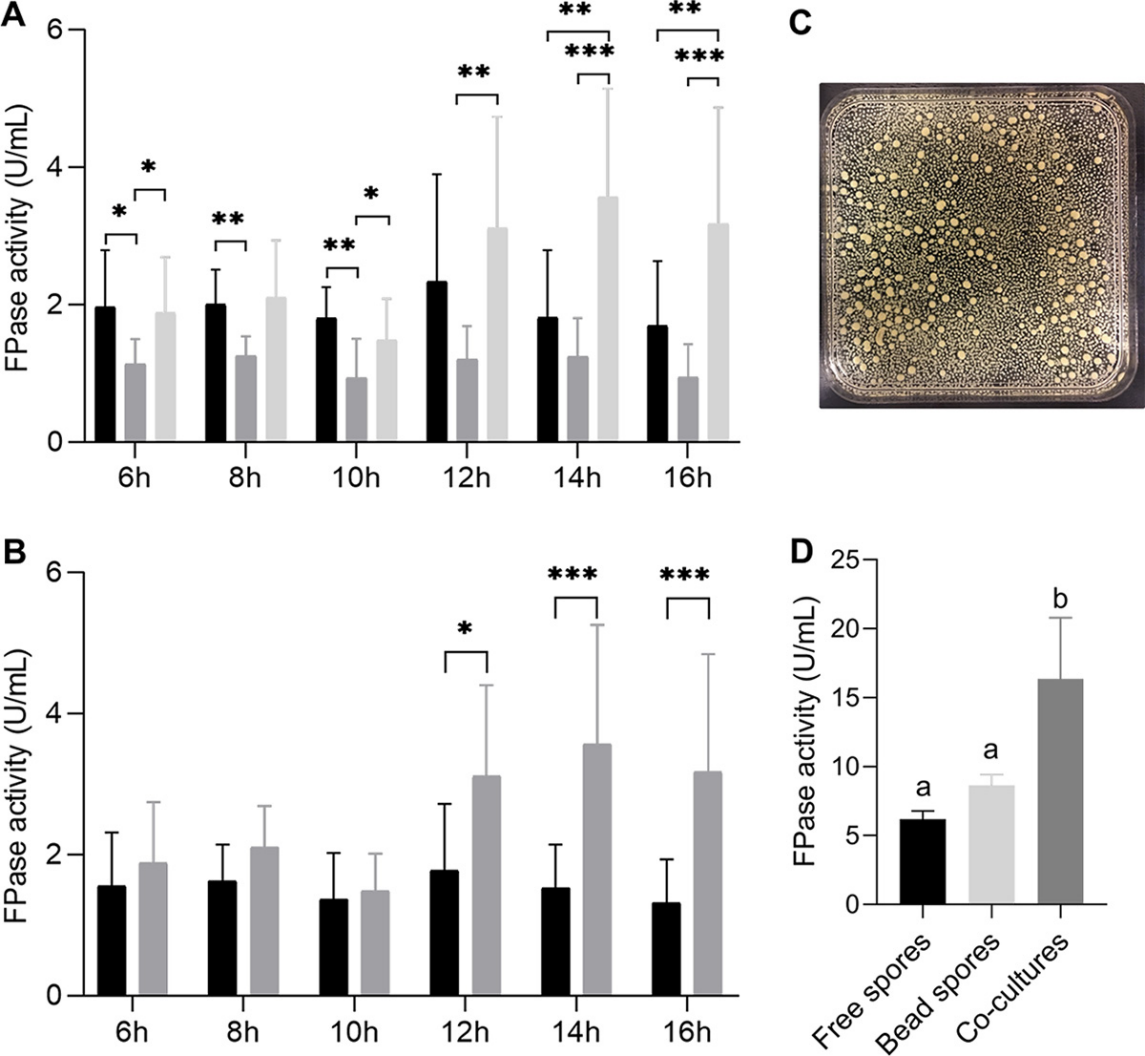
(A) Cellulase activity in culture media of free-spore cultures (black shaded bars) and bead-spore cultures (gray shaded bars) or 1:1 mixtures of these culture media after 48 h of culturing (light gray shaded bars). In (B) the hypothesized cellulase activity in the 1:1 mixed samples (i.e., the average of the free spore and bead spore culture media with the same transfer time) (black shading) is compared with the activity that was actually measured (gray shading). (C) Morphology of cocultures inoculated with pellets/germlings of F12 and B12 cultures (free spores and bead spores cultures that had been cultured for 12 h in liquid shaken TM-X) and grown together for 36 h in static MM-X. (D) Cellulase activity in static cultures of F12, B12 and F12/B12 cocultures after 48 h of culturing (for culturing conditions see C. Statistical analysis of cellulase activity was analyzed by a one-way ANOVA, followed by either a Bonferroni or Dunnett's T3 test for multiple comparisons (A, D) or by either Student’s or Welch's *t* test (B). *, *P* < 0.05; **, *P* < 0.01; and ***, *P* < 0.001 indicate statistical significance. Error bars indicate standard deviations.

Pfam family cellulase was represented by 7 proteins in the set of proteins with a signal sequence in the B6-B16 culture media, while 5 of these proteins were found in the F6-F16 cultures (Table 2). Cellulase activity was compared between the F and B cultures with the same transfer time (e.g., between B6 and F6). Cellulase activity was higher in F6 compared to B6, F8 compared to B8, and F10 compared to B10 (Fig. 6A). Next, cellulase activity was measured after mixing culture media of F and B cultures with the same transfer time (i.e., F6 and B6 were mixed, F8 and B8 were mixed, etc.) in a 1:1 (vol/vol) ratio. Notably, the F12/B12 mixture showed higher activity than the activity that was expected from the individual cultures (Fig. 6B). The same was observed for the F14/B14 and the F16/B16 mixtures. All these mixtures contained medium of cultures with large and small microcolonies. In contrast, the B6/F6 and B8/F8 combinations are made up of media from cultures with only large or intermediate microcolonies and did not show synergistic activity. Together, results show that big and small microcolonies complement each other’s cellulase activity.

Next, it was assessed whether higher cellulase activity was also observed when small and large microcolonies were cocultured. To this end, the F12 and B12 precultures were mixed 1:1 or grown as pure cultures. After a total culturing time of 48 h, cellulase activity of cocultures was not higher compared to the pure F12 and B12 cultures (data not shown). We hypothesized that enzymes of F12 shaken cultures are degraded by proteases in B12 cultures and/or vice versa. Therefore, growth of the F12 and B12 precultures (each originating from the same number of spores) was prolonged for 36 h in static MM-X as pure cultures (total mycelium of the preculture) or by mixing the F12 and B12 cultures (50% of the mycelium of each culture). As expected, the cocultures showed a mix of large and small microcolonies at the end of culturing (Fig. 6C), while the pure cultures showed only small or large microcolonies. Cellulase activity in the medium of the static cultures was significantly higher in the cocultures compared to the pure F12 and B12 static cultures (Fig. 6D). Together, large and small microcolonies can indeed produce a higher cellulase activity when grown together.

### Stress resistance of microcolonies.

**FIG 7:**
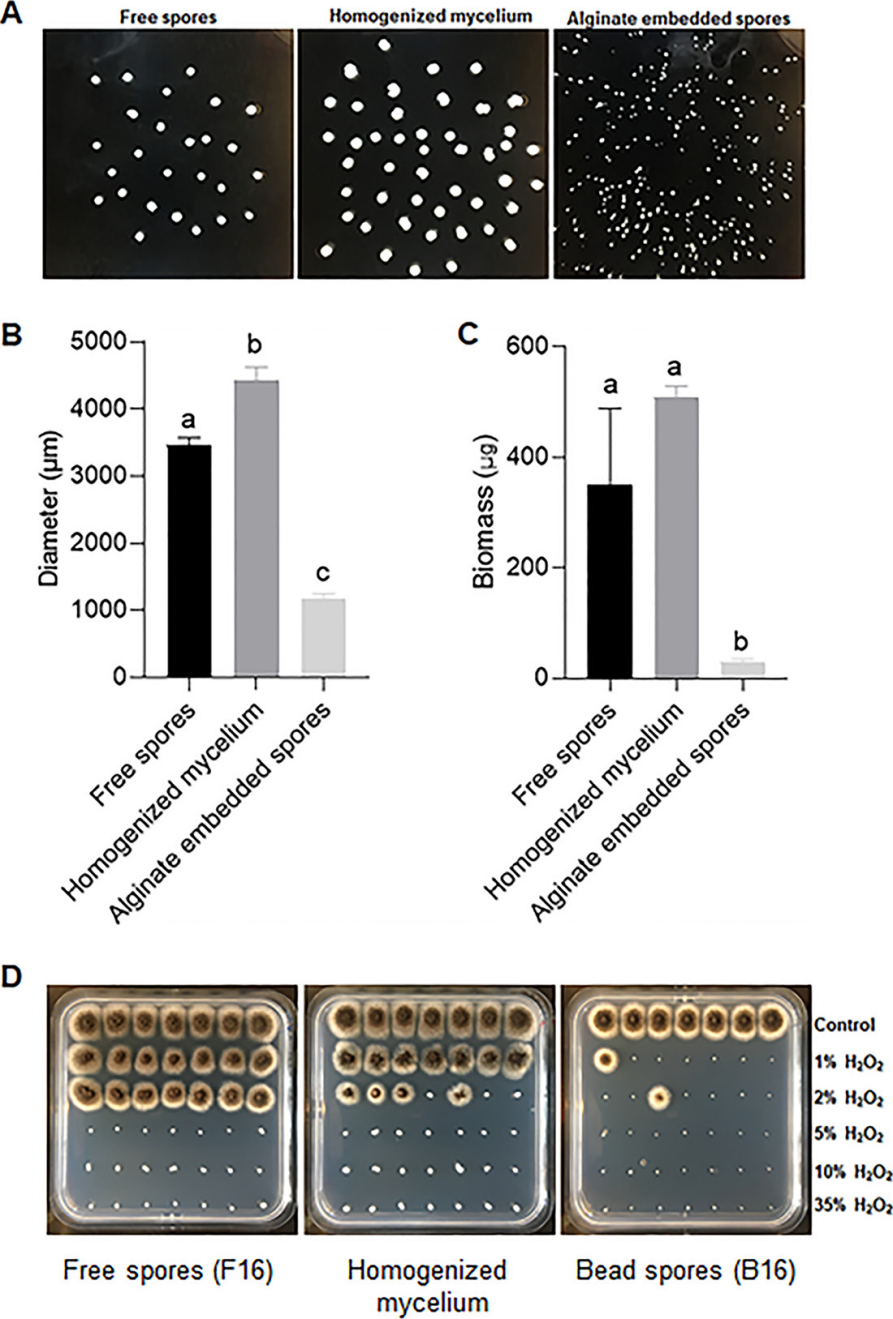
Morphology (A), diameter (B), weight (C) and H_2_O_2_ resistance (D) of microcolonies that had been precultured in TM-X for 16 h and grown for 24 h in MM-X. The precultures had been inoculated with free (F16) or alginate-embedded conidia (B16) or with homogenized mycelium. Individual colonies were transferred to square MMXA plates (7 colonies in each row) after they had been treated 30 min with 1%–35% H_2_O_2_. One-way ANOVA showed statistical significance with Dunnett’s T3 *post hoc* test for diameter and Tukey *post hoc* test for biomass (*P* < 0.05).

Small microcolonies with a diameter of approximately 300 μm cannot be easily handled individually to assess stress resistance. Therefore, it was decided to reduce the concentration of embedded and nonembedded conidia in the culture medium (see Materials and Methods for details). This resulted in large (3411 ± 186 μm; 349.6 ± 139.4 μg) and small (1184 ± 19 μm; 30.2 ± 4.3 μg) microcolonies (Fig. 7). These microcolonies were exposed individually to heat and H_2_O_2_ stress. All small and large microcolonies died after exposure to 60°C for ≥ 30 min or to ≥ 5% H_2_O_2_ for 30 min. A total of 14.3% ± 2% of the small colonies survived a 10 min-exposure at 60°C, while 85.7% ± 11.6% of the large microcolonies resisted this treatment. Similarly, only 9.5 ± 8.2% of the small colonies survived a 30 min treatment with 2% H_2_O_2_, while all big colonies survived. Taken together, large microcolonies are more stress resistant than the small ones.

**FIG 8:**
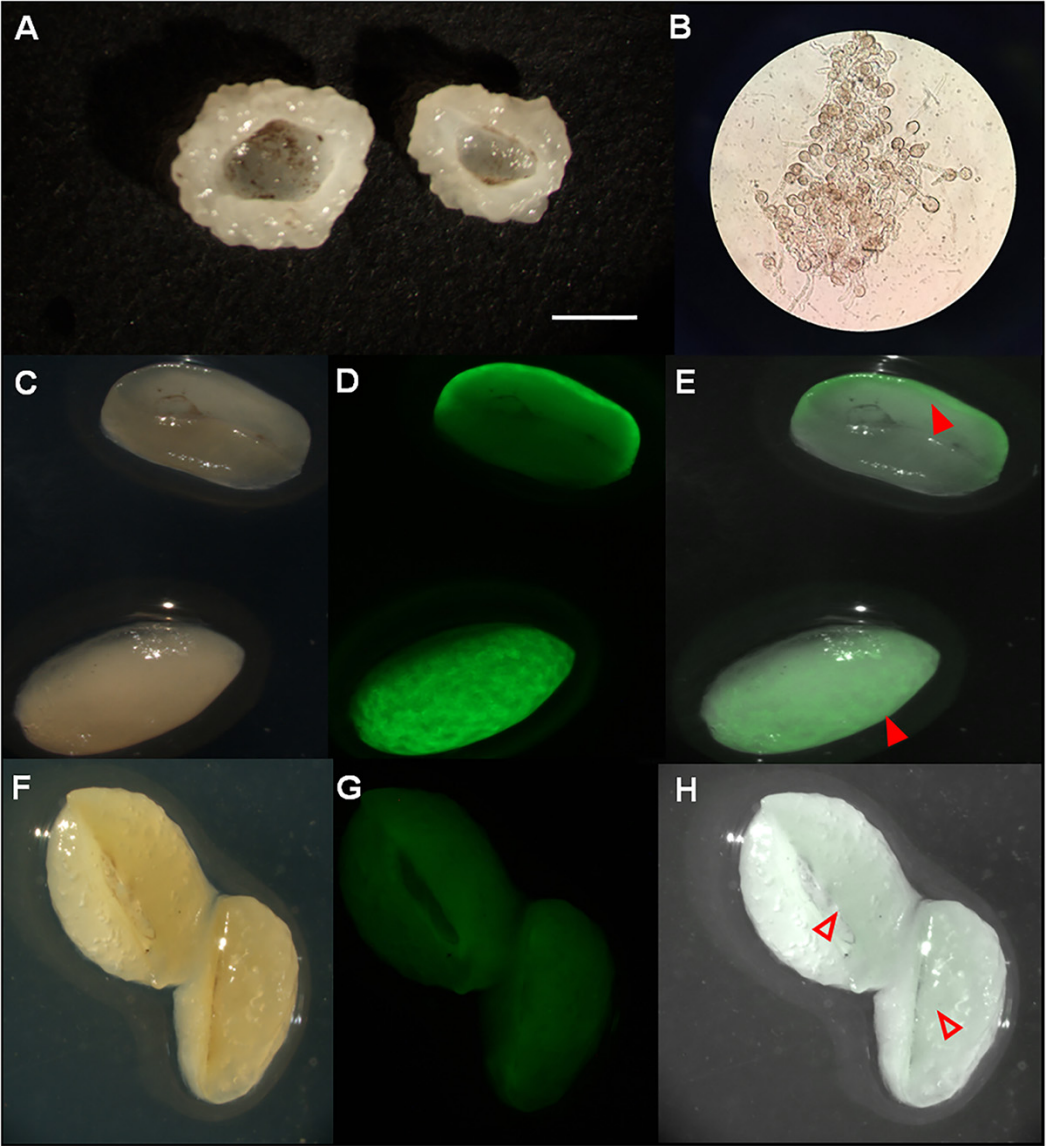
Dissection of a 40-h-old large microcolony resulting from a preculture inoculated with free spores revealed conidia in the center of the pellet as shown by the naked eye (A) and microscopy (B). Brightfield images (C, F) and GFP fluorescence (D, G) and merged pictures (E, H) of large microcolonies of strain AR9#2 that were not-exposed or exposed to 2% H_2_O_2_ for 30 min. Nontreated microcolonies show bright and low fluorescence at the colony periphery and center, respectively. In contrast, H_2_O_2_ treated microcolonies only show low fluorescence in the colony center. Closed and open arrows indicate microcolony periphery and center, respectively. Brightness and contrast in panels F and H have been increased by 40% and decreased by 20%, respectively, to be able to show GFP fluorescence. Bar represents 2 mm (A, C–H).

Large (Fig. 8A) but not small (data not shown) microcolonies showed a black core. Dissecting the large microcolonies revealed the presence of nongerminated conidia in this core (Fig. 8B). Similar structures were not identified in small pellets. Conidia were also observed in large microcolonies when they were grown on glucose or maltose instead of xylose (data not shown). Moreover, they were found both at the end of preculturing (i.e after 16 h) and after the total culturing time of 40 h. To assess if the nongerminated conidia in the center of the large microcolonies were responsible for the higher resistance to environmental stress, a 16 h culture that had been inoculated with free spores was homogenized in a blender and the resulting mycelium homogenate was used to inoculate a preculture. Large microcolonies (4435 ± 330 μm; 509.7 ± 19.2 μg) were formed after transfer of the preculture to MM-X (Fig. 7). As expected, these microcolonies did not contain spores in their center (data not shown). A total of 38.1% ± 8.2% large microcolonies without spores inside their center survived the heat treatment at 60°C for 10 min, while 47.6 ± 8.3% survived the 30 min treatment with 2% H_2_O_2_. These data indicate that the large microcolonies without spores in their center are less stress resistant than those with conidia in the colony core.

**FIG 9:**
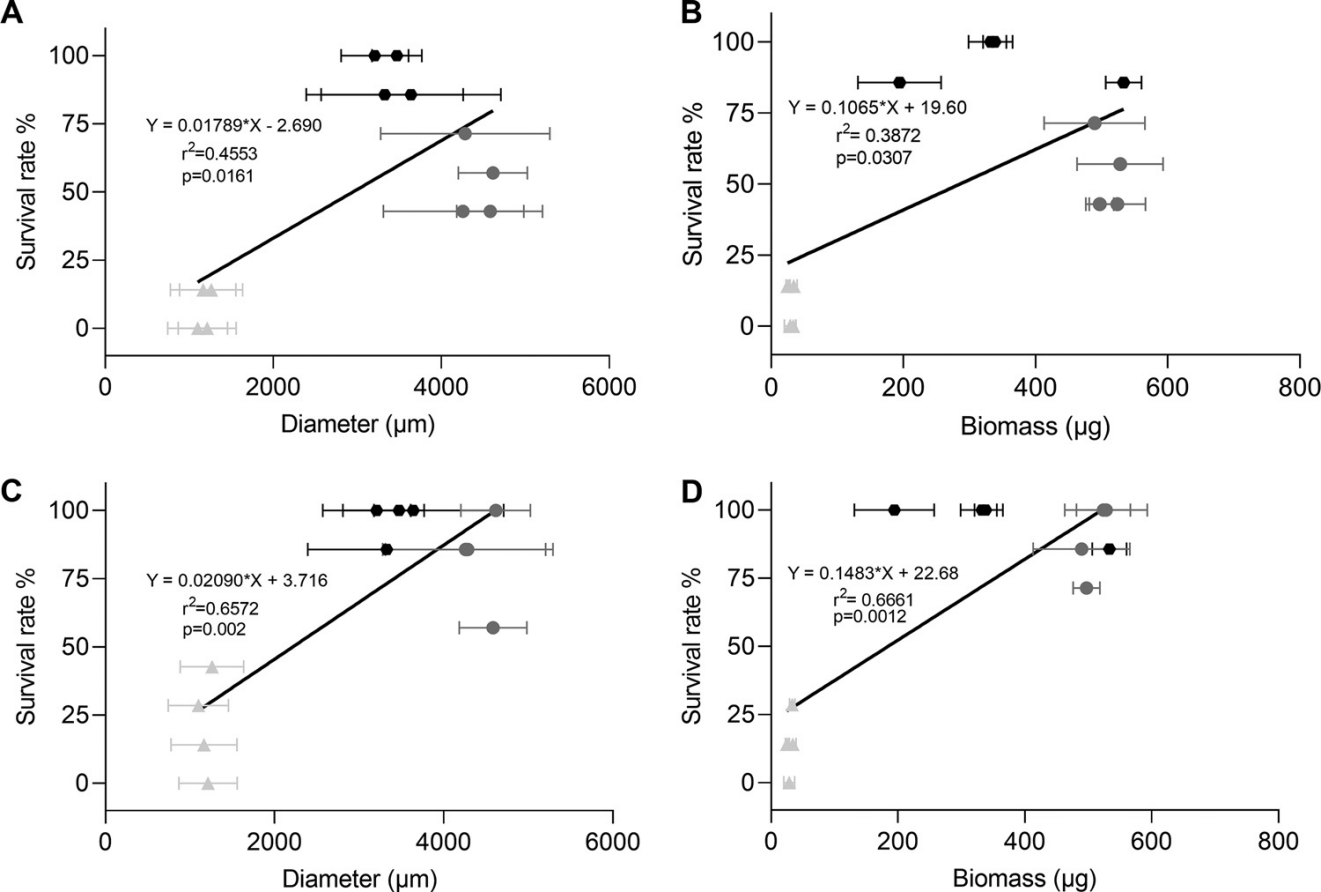
Linear regression analysis of survival after 30 min exposure to 2% H2O2 (A, B) and heat (C, D) and diameter (A, C) and biomass (B, D) of three biological replicates each with seven microcolonies that resulted from alginate embedded conidia (light gray angle), nonembedded conidia (black hexagons) and homogenized mycelium (dark gray circles).

The surface area of microcolonies that had been exposed to heat stress or H_2_O_2_ stress was related to their survival. Linear regression confirmed that both diameter (r^2^ 0.4553, r^2^0.6572) and biomass (r^2^ 0.3872, r^2^0.6661) are related with surival rate to H_2_O_2_ stress (Figure 9A-B). and heat stress (Figure 9C-D), respectively. Taken together, more biomass and larger pellet size contribute to stress resistance.

To study stress resistance in more detail, microcolonies of strain AR9#2 (that expresses *GFP* from the *glaA* promoter) were exposed to H_2_O_2_ stress. Large microcolonies with conidia in their core that had been grown on xylose for a total of 40 h were exposed to 2% H_2_O_2_ for 30 min, after which they were plated for 8 h on maltose containing medium to induce the *glaA* promoter. Bright fluorescence was detected at the periphery of the untreated microcolonies, while low fluorescence was detected in the colony center (Fig. 8C to E). In contrast, the periphery of H_2_O_2_ treated colonies was nonfluorescent, while the colony center was as fluorescent as the center of nontreated colonies (Fig. 8F to H). These results show that the hyphae in the outer zone protect the mycelium within the inner zone from H_2_O_2_ stress.

In the next set of experiments it was assessed whether conidia survive the stress conditions that were used for the microcolonies. Spores from MA.2341 were exposed to heat or H_2_O_2_ directly after harvesting or after a 40 h of incubation in MM at 4°C. In both cases, colonies were only obtained after spotting ≥10^4^ spores. Thus, a single big colony is more resistant than 10^3^ spores.

## DISCUSSION

**FIG 10:**
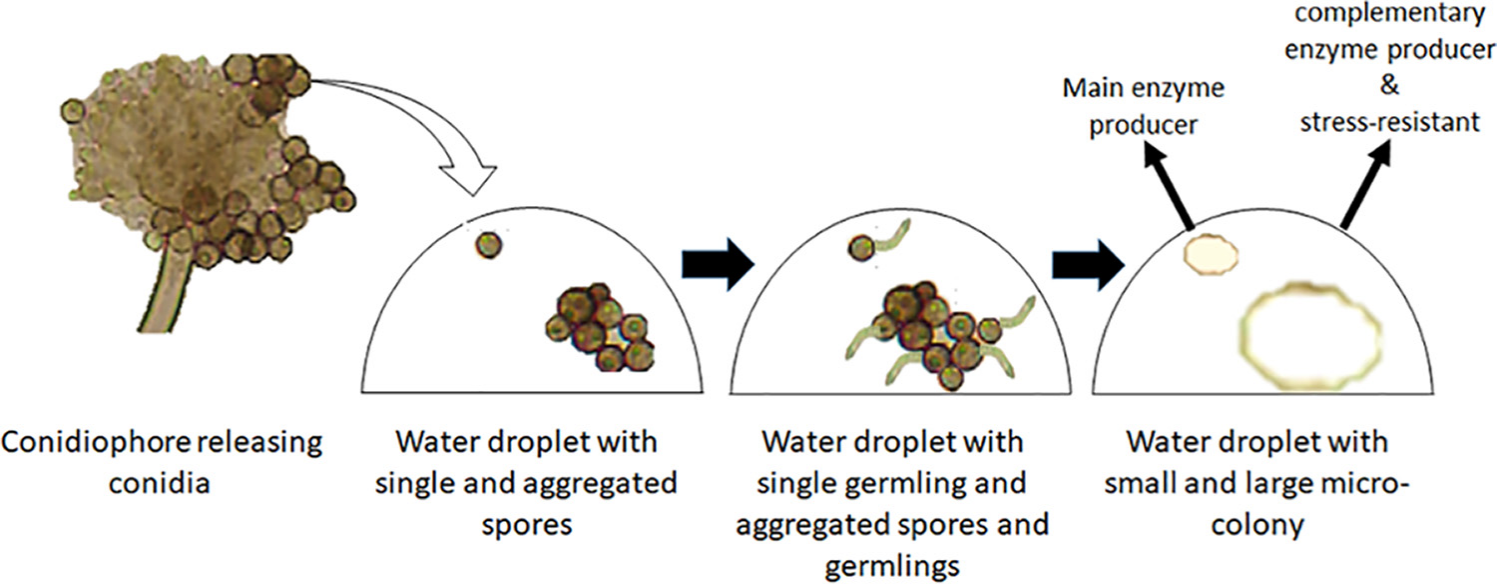
Schematic of formation of small and large microcolonies and their role in an aqueous environment.

A. niger can form microcolonies of different size due to heterogeneity in aggregation of spores and germlings. We here showed that this heterogeneity can be functional and propose that it has actually evolved to form different sized microcolonies that together produce a meta-secretome optimally suited to degrade complex substrates and to promote stress survival (Fig. 10). This functional heterogeneity is not only of interest for the industry to make blends of enzymes (e.g., for biofuel or bioplastic production) but could also play a role in nature for effective nutrient cycling and survival of the fungus.

Previously, morphology of the mycelium of cell factories like A. niger was changed by varying the culture conditions (15). Therefore, changes in yield and secretome composition could have been the result of the changed morphology but also of the changed culture conditions (1, 10). We changed the morphology of the mycelium without changing the culture conditions. To this end, conidia were germinated in alginate beads for different periods, after which the beads were dissolved, followed by transfer of the mycelium to fresh medium to assess protein secretion. Large microcolonies (≥3000 μm) had formed when beads in the precultures had been dissolved after 6 h. This size of the microcolonies was similar to that of cultures that resulted from spores that had not been embedded in alginate (i.e., free spores). In contrast, preculturing in alginate beads for 10 to 16 h resulted in small microcolonies with a diameter of 285 ± 8 μm. These small microcolonies had released 556 different proteins in the culture medium, while this number was 208 in the case of the large microcolonies. In fact, the small microcolonies did not only release a more diverse palette of proteins, they also secreted larger amounts of a set of proteins that were also released by the large microcolonies. From this it can be concluded that smaller pellets are more productive with respect to protein secretion. This may be explained by the fact that production of secreted proteins only occurs at the periphery of the microcolonies in a shell with a relatively constant width (11). Cultures with small microcolonies will have relatively more mycelium in this shell than cultures with the same biomass that form large microcolonies, thus explaining why small microcolonies secrete relatively more protein. Of significance, although the large microcolonies were less effective in protein production, they did release their own unique set of proteins. In fact, cellulase activity of large and small colonies showed synergism when the culture media were combined. We tried to identify regulatory proteins involved in differential protein secretion in large versus small microcolonies. To this end, cellular proteomics was performed (Text S1B). This revealed that the carbonic anhydrase protein AacA and the transcription factor ZtfA were both higher expressed in large microcolonies compared to small microcolonies. Inactivation of their encoding genes did, however, not result in changed biomass, microcolony morphology, or secretome.

About half of the proteins that were released in the medium by the small microcolonies did not have a signal sequence for secretion, while this was only ≤ 15% in the case of large microcolonies. These proteins may be released in the medium by lysis. Aspartyl and serine proteases are regarded as biomarkers of the early stages of autolysis of hyphae (16). These proteins were found in both cultures with large or small microcolonies. Therefore, these proteases are indicative of a basal level of autolysis that occurs in any liquid shaken culture (17). The secretomes of the cultures forming small microcolonies also contained other proteases without signal sequence such as a prolyl oligopeptidase, indicative of a lysis incidence higher than the basal level. It should be noted that microscopic examination did not reveal autolysing hyphae in the cultures. Therefore, proteins without signal sequence may also be released into the medium by a nonclassical pathway. Pore-mediated translocation across the plasma membrane (type I), ABC transporter-based secretion (type II), and autophagosome/endosome-based secretion (type III) are nonclassical secretion systems in eukaryotes that do not make use of a signal sequence for secretion (18). It is not known yet whether any of these types is functional in A. niger, but evidence has been found for the presence of type III nonclassical secretion in yeast (19, 20). Hsp70 is involved in this type of nonclassical secretion (18). Notably, this protein was found in the media of the small microcolonies. Recently, 51 proteins have been suggested to be secreted via the nonclassical pathway in A. niger (21), 31 of them were identified in our database (Text S1C). Of these 31 proteins, 8 proteins did have a signal sequence for secretion. Of the remaining 23 proteins, a total of 13 and 23 were released by large and small microcolonies, respectively. Together, these data suggest that at least some proteins are released by nonclassical secretion.

XlnR is a transcription factor inducing 38 xylanase and cellulase genes (13, 14). The cultures with small microcolonies had released 19 to 23 of these 38 proteins, while those with large microcolonies had released 15 to 18 of these proteins. A total of 6 and 1 XlnR-regulated proteins were exclusively found in the media of small and large microcolonies, respectively. Since the latter microcolonies exclusively produced a cellobiohydrolase, cellulolytic activity was determined in the culture media. Interestingly, mixing the culture media of small and large microcolonies resulted in a higher cellulase activity than expected from the activity in the individual cultures. Similar results were obtained when precultures containing either small or large microcolonies were cocultured under static conditions. These data are of interest from a biotechnology point of view. If one is interested to highly produce a single protein, one would generally opt to grow small microcolonies of uniform size, most preferably with a radius ≤ the width of the peripheral expression zone of this particular gene (11). Yet, if one is interested in a blend of enzymes, for instance to convert agricultural waste into simple sugars for biofuel or bioplastic production, one would use microcolonies with heterogeneous size. Such heterogeneity can be obtained by making blends of precultures with microcolonies of different size. Alternatively, one can use conditions that result in cultures with variable microcolony size.

The synergism in enzyme activity between small and large microcolonies as we observe in liquid cultures raises the question whether this phenomenon also occurs in nature. The surfaces of spores and germlings of A. niger may have evolved to allow that part of the spores and germlings aggregate but not all (Fig. 10). As a result, one would get microcolonies of different size, for instance in a water droplet on a leaf surface. In such a case, these microcolonies would help each other to degrade the plant polymers.

Notably, while the small microcolonies are the main enzyme secretors, the large microcolonies are more resistant to heat and H_2_O_2_. Conidia were found in the core of large pellets (but not in small microcolonies), which most likely originate from the inoculum of the liquid culture. This is based on the observations that conidiophores were absent in the center of the large microcolonies and that the center of large microcolonies resulting from an inoculum of mycelium instead of spores did not contain spores. Together, we propose that only part of the conidia that aggregate during the early phase of culturing germinate. As far as we know, this is the first report of resting conidia in the core of a microcolony. Of interest, the resistance of large microcolonies to heat and H_2_O_2_ was shown to partly result from the conidia in the center of the microcolony. This was based on the finding that large microcolonies resulting from a spore inoculum were more stress resistant than large microcolonies resulting from a mycelium inoculum. It is known that conidia are generally more stress resistant than vegetative hyphae (22), which would explain our findings. Also, the hyphae at the colony periphery have a protective role by preventing damage of hyphae and spores in the colony center. H_2_O_2_ treatment resulted in hyphae at the periphery of large microcolonies that no longer could switch on the *glaA* promoter after transfer to an inducing medium, indicating that these hyphae had been damaged or even killed. In contrast, nontreated colonies showed very bright *glaA* driven GFP fluorescence at the colony periphery. The center of treated and nontreated colonies showed both low fluorescent hyphae, indicating that heat treatment had not damaged the hyphae in this zone.

The reason why some spores in the center of large microcolonies do not germinate is consistent with spore germination studies (23–25). Conidia of A. niger (23, 24) and *Penicillium roqueforti* (25) have a very heterogeneous germination response. For instance, only 20% of the A. niger spores germinate within 14 h in a defined medium with glucose as a carbon source and similar responses were observed when amino acids were used as a carbon source. This heterogeneity was proposed to be a bet hedging strategy to prevent that the whole population would die (23). For instance, when temperature would increase above the cardinal temperature during daytime, the germlings would be killed but not the stress resistant conidia. It is tempting to speculate that heterogeneity in spore germination also has evolved to protect stress survival of microcolonies.

## MATERIALS AND METHODS

### Strain and culture conditions.

Aspergillus niger strains MA.234.1 (transient kusA::amdS; pyrG^+^) (26) and AR9#2 that expresses *GFP* from the *glaA* promoter (27) were grown for 3 days at 30°C on MM (28) with 1.5% agar and 1% glucose. Conidia were harvested with 5 mL saline-Tween (0.8% NaCl, 0.005% Tween 80) or with TM-X (MM with 5 g l^−1^ yeast extract, 2 g l^−1^ Casamino Acids; 25 mM xylose as carbon source, and adjusted to pH 6 with NaOH) (29). The spore suspension was filtered through a syringe with cotton to remove hyphae and the conidia were counted using a hemocytometer.

For secretome analysis, 2 10^7^ spores were introduced in 50 mL TM-X in 250 mL Erlenmeyer flasks (free spore cultures, or F-cultures). Alternatively, the same number of spores were introduced after they had been immobilized in alginate beads with a diameter of 700 μm (bead spore cultures, or B-cultures). To this end, 4% Na-alginate was dissolved overnight in Milli-Q water and sterilized at 121°C for 20 min. The spores were mixed with the alginate solution at a final concentration of 2 10^7^ conidia mL^−1^. The suspension was introduced into a 3% aqueous solution of CaCl_2_.2H_2_O (Sigma-Aldrich, St. Louis, Missouri, United States) with a drop-generating device (30) using 40 cm^3^ min^−1^ pressurized air and 5 cm h^−1^ pump speed. The suspension was mixed with a magnetic stirrer at 300 rpm. As a control, empty beads were added to the culture medium containing free spores. Alternatively, either 1 mL Na-alginate solution or 11 mg CaCl_2_.2H_2_O was added to the culture medium. To dissolve the beads at specific time points, 5 mL 1 M sodium citrate buffer, pH 6.0, was added to the 50 mL cultures. The same amount of buffer was added to the cultures with the free spores and the control cultures. After dissolving the beads, the germlings and/or mycelium were washed twice with 50 mL Milli-Q water with filtering over a filter with 1 μm pores (PluriSelect, Deutscher, Germany). The mycelium was transferred to 100 mL MM-X (MM with 25 mM xylose as a carbon source). After a total period of 48 h of preculturing in TM-X and culturing in MM-X, the mycelium and culture medium were separated by using a 40 μm cell strainer (Corning, 352340, New York, United States). All experiments were carried out using quadruplicates.

To determine stress resistance, microcolonies were produced from conidia or from mycelium. In the former case A. niger was grown in 250 mL Erlenmeyer flasks with 50 mL TM-X. To this end, cultures were inoculated with 2.5 10^6^ spores mL^−1^ that were not taken up or taken up in 700 μm alginate beads (see above) and grown at 30°C and 200 rpm. After 16 h of incubation, the alginate was dissolved by adding 5 mL 1 M Na_3_-citrate buffer (pH 6). The buffer was also added to the control cultures that had been inoculated with nonembedded spores. The mycelium of both cultures was separated from the culture medium by using a 40 μm cell strainer, washed with MM (without carbon source) and transferred to 100 mL MM-X in 250 mL Erlenmeyer flaks. Growth was prolonged for 24 h at 30°C and 200 rpm. To produce microcolonies from mycelium, the latter culture was homogenized for 1 min in a Waring blender, after which 2.5 mL of the homogenate was added to 50 mL TM-X in 250 mL Erlenmeyer flasks. After growing for 16 h, the mycelium was harvested, washed with MM and transferred to 100 mL MM-X and growth was prolonged for 24 h.

### Analyzing microcolony diameter and surface area.

Bright field images of microcolonies were converted to binary images by thresholding. Using the particle analysis tool in ImageJ, microcolonies were automatically segmented using a size > 50,000 square pixel (i.e., 1,000 μm^2^), to get rid of small debris, and a circularity between 0 and 1. The diameter of microcolonies was calculated using the formula $d=\;2\cdot \sqrt{\frac{\mathrm{area}}{\pi }}$ assuming that microcolonies are circular. Bimodality of microcolony diameter within treatments was assessed using a custom script (31). Diameters of the microcolonies belonging to the subpopulation that made up >50% of the total population were analyzed using one-way ANOVA followed by Dunnett’s T3 *post hoc* test for multiple comparisons. Treatments producing similar microcolony diameters was assessed by hierarchical clustering. The impact of the presence of empty beads, alginate, or CaCl_2_ on the diameter of microcolonies grown from free spores was assessed by Student’s or Welch’s *t* test.

### Determination of biomass and protein concentration.

Mycelium was filtered over filter paper and washed twice with distilled water. The mycelium was dried at 60°C and weighed. One-way ANOVA followed by Tukey *post hoc* test was used to assess differences in biomass formation (SPSS 25.0). Protein concentration in the culture medium was measured using Bradford’s method (32). The relation between biomass and microcolony size and between microcolony size and protein release into the medium were assessed by linear regression.

### Resistance to heat and hydrogen peroxide.

Microcolonies (up to a total number of 126) or conidia (up to a total number of 10^6^) were taken up in 20 mL or 100 μL MM-X, respectively, that was preheated at 60°C. Heat stress was stopped by adding a similar volume of MM-X that was precooled at 4°C, after which the microcolonies and conidia were plated on MM-X with 1.5% agar (MM-XA). To this end, conidia were first centrifuged for 1 min at 3000 rpm in an Eppendorf centrifuge, after which they were taken up in 10 μL MM-X. To assess H_2_O_2_ resistance, microcolonies (up to a total number of 126) were transferred to 20 mL MM-X containing up to 35% H_2_O_2_. After 30 min, the pellets were harvested with a 40 μm cell strainer (Corning, 352340, New York, United States), washed with 10 mL MM, and plated on MM-XA in the case of MA234.1 or MM-MA (MM-A with 25 mM maltose as a carbon source) in the case of AR9#2. In the case of conidia, spores (up to 10^6^) of strain MA234.1 were mixed with 1 mL MM containing 1% H_2_O_2_. After 27 min, the suspension was washed three times with MM, each wash followed by centrifugation for 1 min at 3,000 rpm. Conidia were resuspended in 20 μL MM and inoculated in wells of a 12-well-plate filled with MM-XA. Linear regression was used to assess correlation between colony surface area and stress survival rate.

### Fluorescence microscopy.

GFP fluorescence was monitored using a Leica MZ16 stereomicroscope equipped with a mercury lamp, a Leica GFP2 filter set, and a Leica DFC420 C digital camera.

### SDS-PAGE.

Protein contained in 400 μL culture medium was precipitated overnight at −20°C with four volumes precooled acetone. Proteins were pelleted at 4°C at 20,000 g for 30 min and dissolved in 20 μL loading buffer (20% glycerol, 4% SDS, 100 mM Tris-HCl pH 6.8, 0.01% bromophenol blue). Proteins were separated in 12.5% SDS-poly acrylamide gels using TGS buffer (30 g Tris base, 144 g glycine, and 10 g SDS l^−1^). Gels were stained in 0.02% coomassie brilliant blue G-250, 5% Al_2_(SO_4_)_3_(14 to 18)-hydrate, 10% ethanol, and 2% phosphoric acid and destained in 10% ethanol and 2% phosphoric acid (33).

### S-Trap micro spin column protein digestion.

Samples were digested using a S-Trap micro spin column (Protifi) following the manufacture’s protocol. Briefly, samples were mixed 1:1 with lysis buffer (10% SDS, 1 tablet cOmplete mini Protease inhibitor 10 mL^−1^ (Roche), 100 mM TEAB, pH 8.5) and centrifuged for 8 min at 13,000 g. Protein in the supernatant was reduced with 4 mM DTT for 30 min at 56°C, alkylated with 8 mM iodoacetamide for 20 min at room temperature and acidified to 1% phosphoric acid. After a 1:7 dilution with S-Trap binding buffer (90% MeOH, 100 mM TEAB, pH 7.1), samples were loaded onto the spin column and passed through the column at 2,000 g, followed by 3 washes with S-trap binding buffer. Samples were digested with 3 μg trypsin (Promega, WI, USA) for 1 h at 47°C, eluted with 40 μL each of 50 mM TEAB, 0.2% formic acid, and 50% acetonitrile in 0.2% formic acid. Samples were dried in a vacuum concentrator and resuspended in 2% formic acid prior to UHPLC-MS/MS.

### Data acquisition.

Data were acquired using an UHPLC 1290 system coupled to an Orbitrap Q Exactive HF-X mass spectrometer (Thermo Scientific). Peptides were trapped on a column (Maisch Reprosil C18, 3 μm, 2 cm × 100 μm), after which they were separated on an analytical column (Agilent Poroshell EC-C18, 278 μm, 40 cm × 75 μm) using a gradient of 39 min at a column flow of 300 nl min^−1^. Trapping was performed at 5 μL min^−1^ for 5 min in solvent A (0.1% formic acid in water) and eluted using as gradient; 13 to 40% solvent B (0.1% formic acid in 80% acetonitrile) in 35 min, 40 to 100% in 3 min and 100% solvent B for 1 min. Full scan MS spectra from *m/z* 375 to 1600 were acquired at a resolution of 60,000 at *m/z* 400 after accumulation to an automatic gain control target value of 3e6 ions. Up to 15 most intense precursor ions were selected for fragmentation. HCD fragmentation was performed at normalized collision energy of 27% after the accumulation to a target value of 1e5. MS/MS was acquired at a resolution of 30,000.

### Proteomics data analysis.

Raw data were analyzed with MaxQuant software (version 1.6.8.0) using label-free quantification (34). A false discovery rate (FDR) of 0.01 for proteins and peptides and a minimum peptide length of 7 amino acids were required. MS/MS spectra were searched against the database using the Andromeda search engine. Trypsin allowing N-terminal cleavage to proline was selected for enzyme specificity. Cysteine carbamidomethylation was selected as fixed modification, while protein N-terminal acetylation and methionine oxidation were selected as variable modifications. Up to two missed cleavages were allowed, as well as an initial mass deviation of precursor ion ≤ 7 ppm, an a mass deviation for fragment ions of 0.05 Da. Protein identification required one unique peptide to the protein group and match between run was enabled. In all cases, at least 2 technical replicates and 4 biological replicates were used, the latter showing R^2>0^.97. All correction analyses were carried out with Perseus software Version 1.6.10.0. Proteins were considered present when they had been identified in at least 3 out of 4 biological replicates. Pfam overrepresentation and signal peptide analysis were done as described (35, 36).

### cDNA synthesis and quantitative PCR analysis.

cDNA was synthesized from total RNA using the QuantiTect reverse transcription kit (Qiagen, Hilden, Germany). Quantitative PCR (QPCR) was performed by using ABI Prism 7900HT SDS and SYBR green chemistry (Applied Biosystems, Carlsbad, CA) and primers as indicated in Table S1. The primer pairs had an amplification efficiency of 91.5% −112.5%. Expression of genes was related to levels of actin mRNA using the 2^-ΔΔCT^ method using QuantStudio Real-Time PCR Software (Applied Biosystems, Carlsbad, CA), setting the relative expression of the F12 sample at 1. Data were analyzed by *t* test comparing F12 and B12 samples after 12 and 48 h.

10.1128/mbio.00870-22.1 TABLE S1 Primers used in this study. Download Table S1, DOCX file, 0.01 MB. Copyright © 2023 Lyu et al. 2023 Lyu et al. https://creativecommons.org/licenses/by/4.0/ This content is distributed under the terms of the Creative Commons Attribution 4.0 International license.

### Cellulase activity assay.

Cellulase activity was quantified using the filter paper activity assay (FPase) (37). To this end, 7-mm diameter circles of Whatman No. 1 filter paper were incubated with 60 μL culture medium for 24 h at 50°C. This was followed by a 5 min incubation at 95°C after adding 120 μL DNS (10 g L^−1^ 3,5-dinitrosalicylic acid, 400 g L^−1^ KNa-tartrate, and 16 g NaOH l^−1^). Aliquots of the samples (100 μL) were transferred to the wells of a flat-bottom plate and the A_540_ was determined using a Synergy HTX Microplate Reader (BioTek, Winooski, VT, USA). Calibration curves were made using different concentrations of glucose. A unit of cellulase activity was defined as 1 μmol glucose released in 1 min. Cellulase activity within the culture media of B and F cultures that had been transferred to MM-X at the same time point (e.g., B6 and F6) were compared to a 1:1 mixture of these media by one-way ANOVA, followed by either a Bonferroni or Dunnett's T3 test for multiple comparisons. Similar statistical analysis was done when large and small microcolonies were cultured individually or in cocultures. Additionally, hypothesized cellulase activity of 1:1 mixed medium, defined as the mean cellulase activity of medium originating from B and F cultures, were compared to the actual cellulase activity in the mixtures by either Student’s or Welch's *t* test.
