# Supplementary material for: Heterogeneity in Spore Aggregation and Germination Results in Different Sized, Cooperative Microcolonies in an Aspergillus niger Culture
Source: mBio. 2023 Jan 11;14(1):e00870-22. doi: 10.1128/mbio.00870-22 (PMC9973262; doi:10.1128/mbio.00870-22)
Supplement: FIG S1 [file mbio.00870-22-s0008.docx]

**
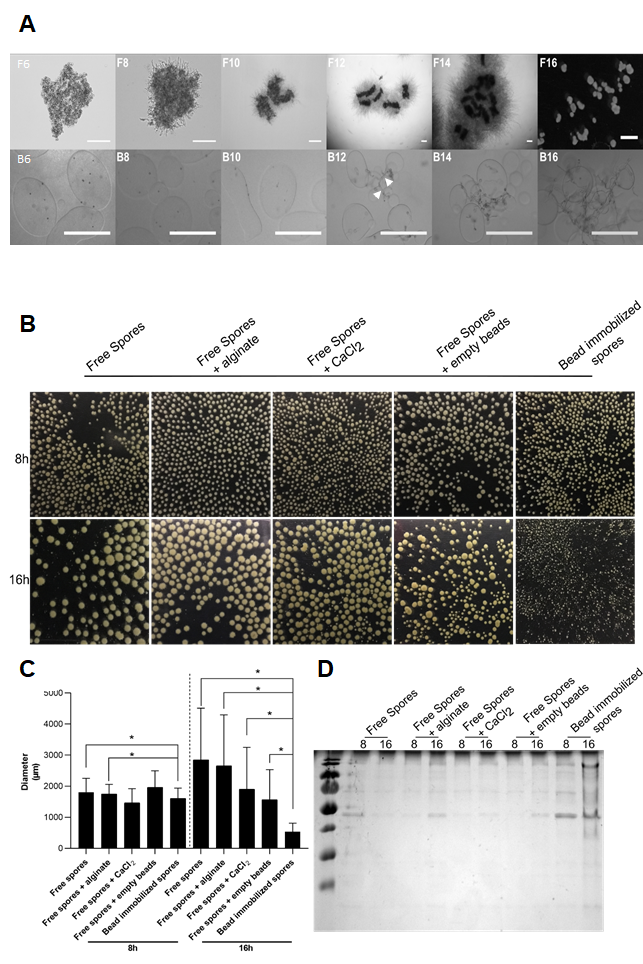
**

**Supplemental Figure 1.** Morphology of spores and germlings of 6-16 h free spores (F6-16) and bead spores (B6-B16) in TM-X liquid shaken cultures (**A**). Bar represents 100 µm in F6-F14 and B6-B16 and 5000 µm in F16. Free spores are clustered at 6 h and had started to form germlings at 8 h. Aggregation of spores and germlings increased in time. Spores embedded in beads did not aggregate and had formed germ tubes at 12 h (indicated by arrow heads). Morphology of mycelium (**B, C**) and proteins released in the culture medium (**D**) from free spore cultures F8 and F16, to which either or not empty beads, alginate or CaCl_2_ had been added. Bead spore cultures B8 and B16 served as a control. The beads had been dissolved after 8 h or 16 h of pre-culturing in TM-X and mycelium of the B and F pre-cultures was transferred to MM-X for 40 and 32 h, respectively, to have a total culturing time of 48 h. Statistical analysis in **C** was performed with a t-test or Welch’s t-test.
