# Supplementary material for: Heterogeneity in Spore Aggregation and Germination Results in Different Sized, Cooperative Microcolonies in an Aspergillus niger Culture
Source: mBio. 2023 Jan 11;14(1):e00870-22. doi: 10.1128/mbio.00870-22 (PMC9973262; doi:10.1128/mbio.00870-22)
Supplement: FIG S2 [file mbio.00870-22-s0009.docx]

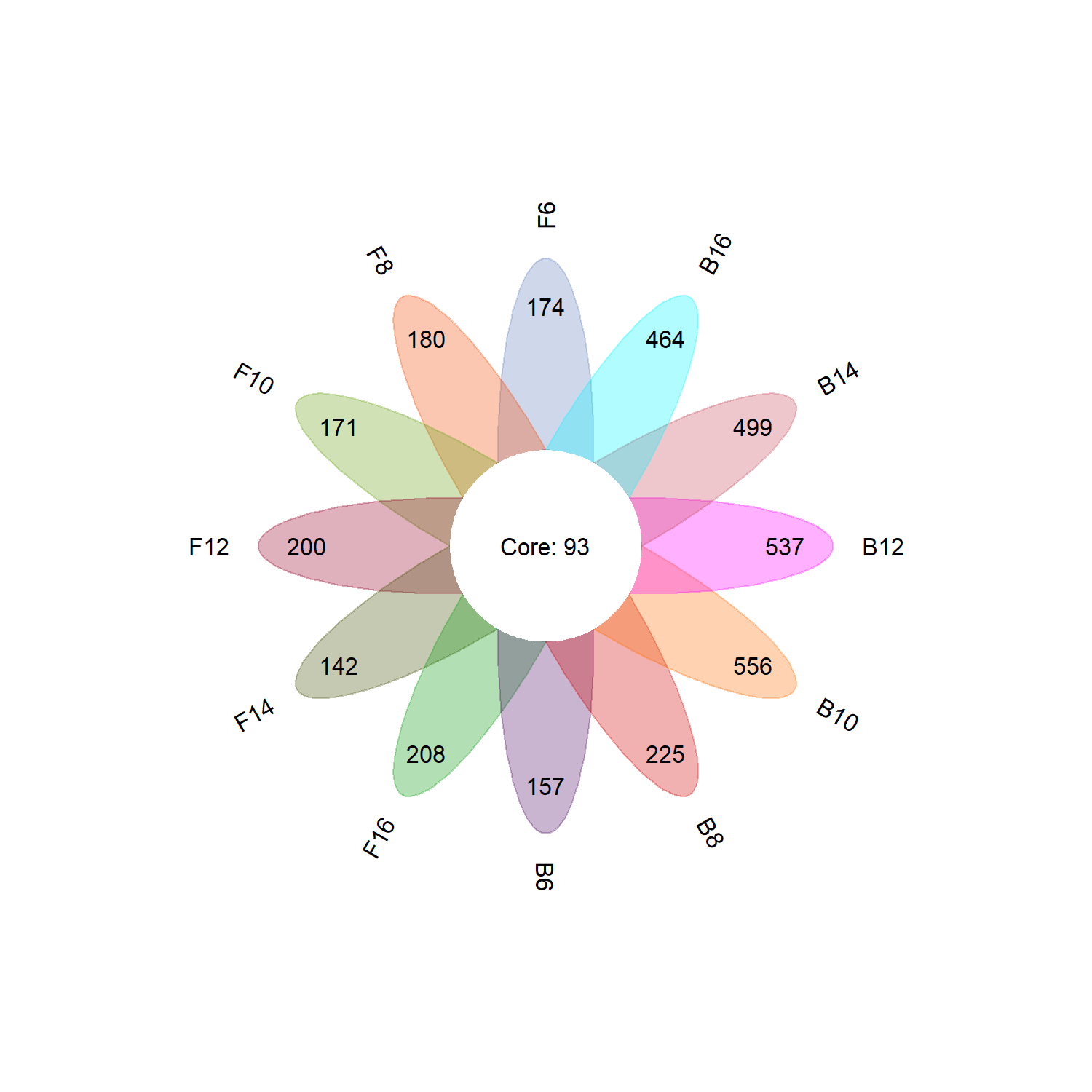

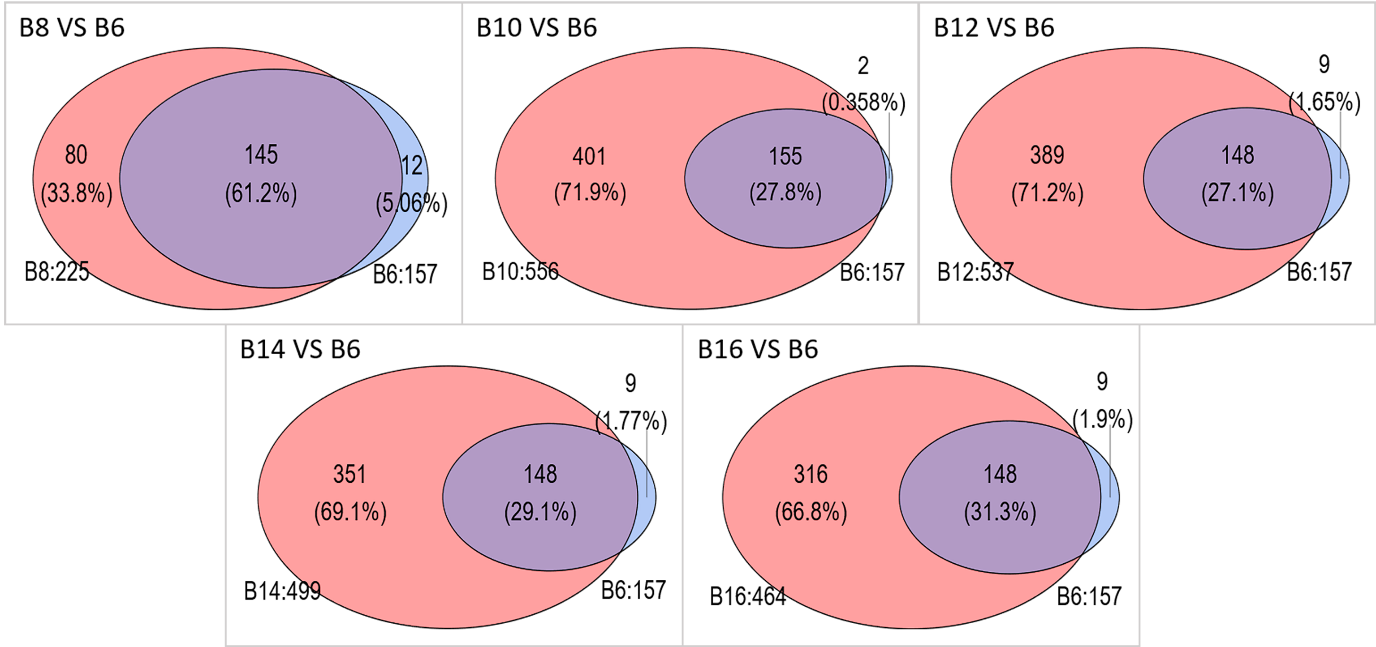

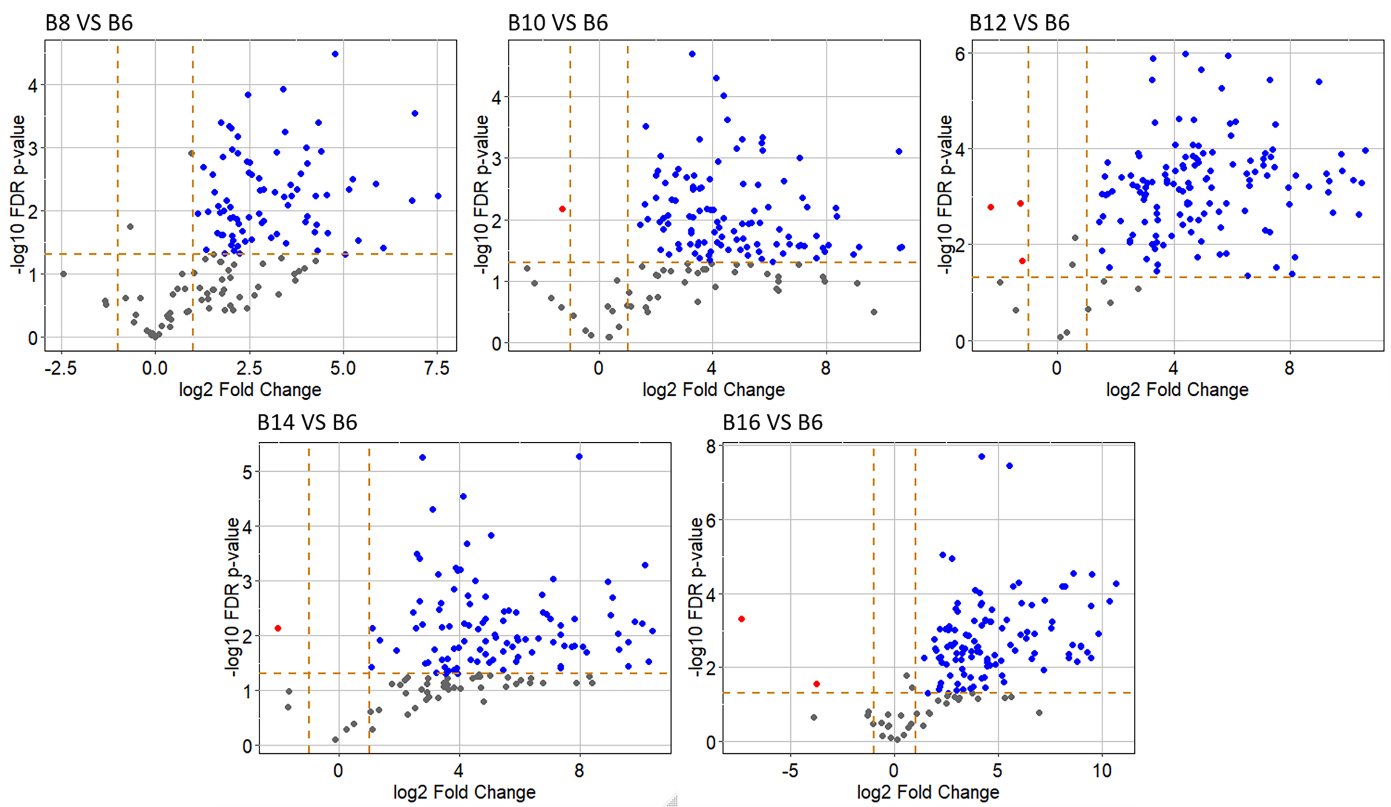


**A**

**B**

**C**

**Supplemental Figure 2.** (**A**) Flower plot of proteins from free spore (F) and bead spore (B) cultures. The beads had been dissolved after 6-16 h of pre-culturing (indicated by the numbers following B) and culturing was extended for 42-32 h after transfer of the mycelium to have a total culturing time of 48 h. Total number of proteins not being part of the core are indicated in the leaves of the flower plot. (**B, C**) Pairwise comparison of number (**B**) and quantity (**C**) of proteins from bead spore B6 cultures with B8, B10, B12, B14 and B16 cultures. Proteins were analyzed after a total culturing time of 48 h. The number following B indicates the time of pre-culturing in hours. Red and blue dots indicate proteins that were ≥2 fold up- or down-regulated in B6 cultures when compared to the other B cultures.
