# Supplementary material for: Heterogeneity in Spore Aggregation and Germination Results in Different Sized, Cooperative Microcolonies in an Aspergillus niger Culture
Source: mBio. 2023 Jan 11;14(1):e00870-22. doi: 10.1128/mbio.00870-22 (PMC9973262; doi:10.1128/mbio.00870-22)
Supplement: TEXT S1 [file mbio.00870-22-s0010.docx]

**sUPPLEMENTAL tEXT 1a**

Protein family (Pfam) analysis was performed to assess the functions of proteins in the secretomes with (Table 2, Supplemental Table 6A) and without (Table 2, Supplemental Table 6B) predicted signal sequences. The F6-F16 cultures that all formed large micro-colonies showed 7-13 Pfam families in the set of proteins with a signal sequence and 4-6 Pfam families in the set without signal sequences (Table 2, Supplemental Table 6 A,B). Glucanosyltransferase was the Pfam family with most members (i.e. 6 representatives) in a specific culture in the set of proteins with signal sequence. B6 and B8 cultures that formed large and intermediate size micro-colonies showed a similar number of Pfam families in the set of proteins with signal sequence (6 and 11, respectively) and without signal sequence (6 and 9, respectively) when compared to the F6-F16 cultures (Table 2, Supplemental Tables 6 AB). On the other hand, 15-19 and 15-22 Pfam families were found in the sets of proteins with and without signal sequence, respectively, in the media of the B10-B16 cultures that form small micro-colonies, which was higher than that found in the F6-F12 cultures. Glucanosyltransferase and glycosylhydrolase family GH43 (each 6 proteins) and carboxypeptidase and cellulase (each 7 proteins) were the Pfam families with most members in the set of proteins with signal sequence within specific B10-B16 cultures. Together, cultures with small micro-colonies have more Pfam families in their secretomes when compared to cultures with intermediate or large micro-colonies.

**Supplemental Text 1B**

Cellular proteomics was performed to identify proteins that are differentially expressed in large and small micro-colonies. Two genes encoding such proteins were deleted but their phenotype was not different from the wild-type strain.

**Material and Methods**

*Cellular proteomics*

Cellular proteomics was performed as described (Lyu, 2021). The R package DESeq2 was used to calculate differential protein abundance. The Benjamini-Hochberg correction was used to reduce false positives at p-value ≤ 0.05.

**Table 1.** sgRNA sequences used in this study

| sgRNA | Sequence |
| --- | --- |
| Sg25 | ATTGCCGACGAGAATCCCCA |
| Sg26 | GGGGACAGCAGTGTTAGGTG |
| Sg27 | TCTGGTCAGGCACTCCCGCG |
| Sg28 | CTCCACCAATCAGCGATGCG |

*Gene inactivation*

Genes were inactivated as described (Lyu, 2021). SgRNAs sg25 and sg26 were used for inactivation of the carbonic anhydrase gene *caaA* (ATCC64974_10520), while sg27 and sg28 were used to inactivate the Zn(2)-C6 fungal-type domain transcription factor gene *ztfA* (ATCC64974_49970) (Table 1). These sgRNAs produce a double cut 130 and 138 bp before the start codon of *caaA* and *ztfA* and 264 and 74 bp after the stop codon of these genes, respectively. The sgRNAs were expressed under control of the RNA pol III promoter and the *trpC* terminator in plasmid pMT12.5 (Table 2). This plasmid is a derivative of pFC332 that contains the *cas9* gene, a hygromycin resistance cassette (Nodvig et al., 2018; van Leeuwe et al., 2019; Table 2), and the *GFP* gene under control of the RNA pol III promoter and the *trpC* terminator. The sgRNAs (primer pair 1/2 and 3/4 for up- and down-cut sgRNA of *caaA*, 8/9 and 10/11 for up- and down-cut sgRNA of *ztfA*, respectively; Table 3) were cloned in pMT12.5 by digestion with BtsI that removes the *GFP* coding sequence. This resulted in plasmids pJL025, pJL026, pJL027 and pJL028, respectively (Table 2). Plasmid pair pJL025 and pJL026 and pJL027 and pJL028 were introduced in *A. niger* MA234.1 combined with the repair oligos RO001 and RO002 for *caaA* and *ztfA* (Table 3), respectively. Gene deletions were confirmed by PCR using genomic DNA of strain MA234.1 and the transformants as template and the primers described in Table 3. Primer pair 6/7 was designed to give a 2571 bp fragment in the wild-type but a 1001 bp fragment in the *caaA* deletion strain, while primer pair 13/14 was designed to give a 2444 bp fragment in the wild-type but a 916 bp fragment in the *ztfA* deletion strain.

**Table 2.** Plasmids used in this study

| Plasmid | Description | Reference |
| --- | --- | --- |
| pFC332 | contains *cas9,* a hygromycin resistance cassette and a PacI site for introducing the sgRNA | (Nodvig et al., 2018; Seekles et al., 2021) |
| pMT12.5 | Derivative of pFC322 in which the RNA pol III promoter and the *trpC* terminator flank the GFP gene | Unpublished |
| pJL025 | Derivative of pMT12.5 containing the upstream *caaA* sgRNA | This study |
| pJL026 | Derivative of pMT12.5 containing the downstream *caaA* sgRNA | This study |
| pJL027 | Derivative of pMT12.5 containing the upstream *ztfA* sgRNA | This study |
| pJL028 | Derivative of pMT12.5 containing the downstream *ztfA* sgRNA | This study |

**Table 3.** Primers used in this study

|  | Primer name | Sequence |
| --- | --- | --- |
| 1 | caaA.Uc.Fwd | ATTGCCGACGAGAATCCCCAGT |
| 2 | caaA.Uc.Rev | TGGGGATTCTCGTCGGCAATGA |
| 3 | caaA.Dc.Fwd | GGGGACAGCAGTGTTAGGTGGT |
| 4 | caaA.Dc.Rev | CACCTAACACTGCTGTCCCCGA |
|  | RO001 | TGCACTGTTTGCATGGTTGGGGGTGAGCATTGCCGACGAGAATCCTAGGGATAACAGGGTAATGGGGGGTGTGGAGTCAAGGAATCAATAGTAGCAGTGAGGAAGAAAGTAGA |
| 6 | caaA.Cf.Fwd | CCCTGTGACTTTACAGACTA |
| 7 | caaA.Cf.Rev | GAGAGATAGGTTGTAGTCGA |
| 8 | ztfA.Uc.Fwd | TCTGGTCAGGCACTCCCGCGGT |
| 9 | ztfA.Uc.Rev | CGCGGGAGTGCCTGACCAGAGA |
| 10 | ztfA.Dc.Fwd | CTCCACCAATCAGCGATGCGGT |
| 11 | ztfA. Dc.Rev | CGCATCGCTGATTGGTGGAGGA |
|  | RO002 | ATATAGCTGGCTTCCGCATTAGGATTAGTCTGGTCAGGCACTCCCTAGGGATAACAGGGTAATGGGGGGCGGGGAACGTAGATGCCTGGATGCTGAGGCGCAGCCTCGTCGTG |
| 13 | ztfA.Cf.Fwd | CGATGAATGCTCCTGGACCC |
| 14 | ztfA.Cf.Rev | TGGTGCAGGAGCATGGACAG |

**Results**

Cellular proteomics of F12 and B12 cultures was performed to assess which proteins are up- and down-regulated in large and small micro-colonies, respectively. SDS PAGE showed that differences in the secretome started to be visible 18 h after culturing (12 h pre-culturing in TM-X followed by 6 h culturing in MM-X) and these became more pronounced in time (Figure 1A). Therefore, it was decided to perform cellular proteomics after 18, 24, and 48 h of culturing.


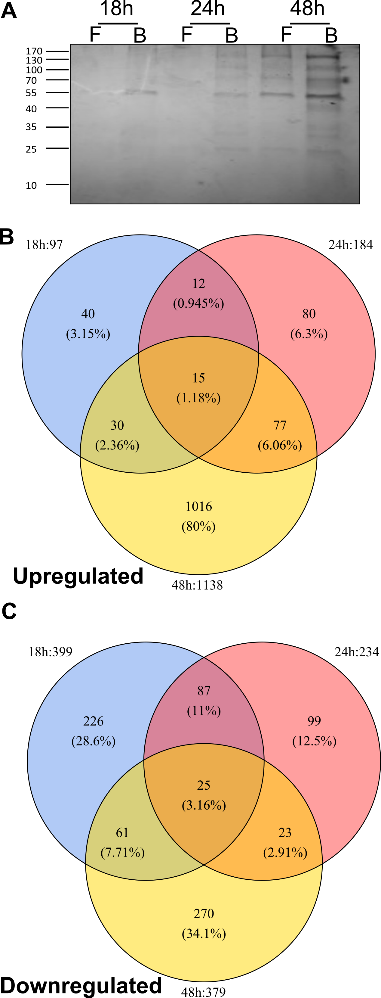


**Figure 1.** Protein profiles of the culture medium of F12 and B12 cultures that had formed large and small micro-colonies, respectively, 18, 24 and 48 h after transfer to MM-X (A). Venn diagrams showing proteins that are significantly up- (B) and down- (C) regulated in B12 versus F12 cultures at all time points (i.e. after 18, 24, and 48 h of culturing).

**Table 4.** Protein family analysis of the proteins that were up- and down-regulated in cultures of small micro-colonies compared to large micro-colonies at time points 18, 24 and 48 h.

| Protein ID | PFAM | Annotation | |
| --- | --- | --- | --- |
| **Significant upregulated proteins** | | |  |
| ATCC64974_104630 | PF00248 | Aldo/keto reductase family | |
| ATCC64974_109280 | PF00107 | Zinc-binding dehydrogenase | |
| ATCC64974_109480 | PF05362 | Lon protease (S16) C-terminal proteolytic domain | |
| ATCC64974_14700 | PF01055 | Glycosyl hydrolases family 31 | |
| ATCC64974_26850 | PF13714 | Phosphoenolpyruvate phosphomutase | |
| ATCC64974_27560 | PF01408 | Oxidoreductase family, NAD-binding Rossmann fold | |
| ATCC64974_35130 | PF00568 | WH1 domain | |
| ATCC64974_47770 | PF18404 | Glucosyltransferase 24 | |
| ATCC64974_63220 | PF00106 | short chain dehydrogenase | |
| ATCC64974_64650 | PF00561 | alpha/beta hydrolase fold | |
| ATCC64974_80810 | PF12110 | Nuclear protein 96 | |
| ATCC64974_95010 | PF00171 | Aldehyde dehydrogenase family | |
| ATCC64974_96990 | PF00083 | Sugar (and other) transporter | |
| ATCC64974_7980 | PF01042 | Endoribonuclease L-PSP | |
| ATCC64974_28350 | PF01077 | Nitrite and sulphite reductase 4Fe-4S domain | |
| **Significant downregulated proteins** | | |  |
| ATCC64974_10520 | PF00484 | Carbonic anhydrase | |
| ATCC64974_108150 | PF00203 | Ribosomal protein S19 | |
| ATCC64974_110190 | PF02114 | Phosducin | |
| ATCC64974_12930 | PF00270 | DEAD/DEAH box helicase | |
| ATCC64974_1450 | PF00076 | RNA recognition motif. (a.k.a. RRM, RBD, or RNP domain) | |
| ATCC64974_17780 | PF13344 | Haloacid dehalogenase-like hydrolase | |
| ATCC64974_25810 | #N/A | #N/A | |
| ATCC64974_26440 | PF11563 | Protoglobin | |
| ATCC64974_3500 | PF13041 | PPR repeat family | |
| ATCC64974_37420 | PF00962 | Adenosine/AMP deaminase | |
| ATCC64974_3930 | PF00026 | Eukaryotic aspartyl protease | |
| ATCC64974_50990 | PF00022 | Actin | |
| ATCC64974_51730 | PF00735 | Septin | |
| ATCC64974_56970 | PF05046 | Mitochondrial large subunit ribosomal protein (Img2) | |
| ATCC64974_64890 | PF01937 | Protein of unknown function DUF89 | |
| ATCC64974_65560 | PF01912 | eIF-6 family | |
| ATCC64974_670 | PF08611 | Fungal protein of unknown function (DUF1774) | |
| ATCC64974_73840 | PF08491 | Squalene epoxidase | |
| ATCC64974_77310 | PF03795 | YCII-related domain | |
| ATCC64974_79720 | PF06201 | PITH domain | |
| ATCC64974_89170 | PF02752 | Arrestin (or S-antigen), C-terminal domain | |
| ATCC64974_97840 | PF00155 | Aminotransferase class I and II | |
| ATCC64974_67810 | #N/A | #N/A | |
| ATCC64974_35260 | PF01849 | NAC domain | |
| ATCC64974_32430 | PF02826 | D-isomer specific 2-hydroxyacid dehydrogenase, NAD binding domain | |

A set of 97 and 399 proteins were significantly up- and down-regulated in small and large micro-colonies, respectively, after 18 h of culturing, while these values were 184 and 234 after 24 h and 1138 and 379 after 48 h of culturing. Among these proteins, 15 and 25 were up- and down-regulated at all three time points in small when compared to large micro-colonies (Figure 1BC, Table 4). The carbonic anhydrase gene *caaA* (ATCC64974_10520) was identified as one of the significantly downregulated proteins in small micro-colonies. Its encoding protein catalyzes the reversible conversion of CO_2_ to HCO_3_^-^ and as such is involved in the formation of the second messenger cAMP. Also, *caaA* is predicted as co-expressed with the α-1,3-glucan synthase gene *agsE* (ATCC64974_10510) with a correlation of 0.85 (fungiDB). Indeed, inactivation of *agsE* resulted in small micro-colonies and reduced expression of *caaA* (Lyu, 2021). This raised the question what the effect would be of inactivation of *caaA* on micro-colony morphology and the secretome. In addition, a transcription factor was selected that was differentially expressed at all three time points (this in contrast to all other differentially expressed transcription factors; Table 5). This Zn(2)-C6 fungal-type domain-containing transcription factor protein ZtfA (ATCC64974_49970) was higher expressed in large micro-colonies (resulting from free spores) at time points 18 h and 24 h, while it was downregulated at timepoint 48 h (only identified in 2 out of 4 replicates in B48, therefore not regarded as expressed at this time point). Both *caaA* and *ztfA* deletion strains did not show any phenotype with respect to biomass, micro-colony morphology and secretome (Figure 2).

**Table 5.** Transcription factor analysis of the proteins which identified as significantly up- or downregulated in small micro-colonies compared to large micro-colonies at least in different timepoint. Transcription factors were identified using the Fungal transcription factor database (FTFD, <http://ftfd.snu.ac.kr/intro.php>) (Park et al., 2008).

| Protein ID | Annotation |
| --- | --- |
| **B18vsF18 up** | -- |
| **B18vsF18 down** |  |
| ATCC64974_49970 | Fungal Specific TF; Zn(2)-C6 fungal-type domain-containing protein |
| ATCC64974_69840 | CCR4-Not complex component, Not1; regulation of translation |
| **B24vsF24 up** |  |
| ATCC64974_96430 | Fungal Specific TF; CN hydrolase domain-containing protein |
| **B24vsF24 down** |  |
| ATCC64974_49970 | Fungal Specific TF; Zn(2)-C6 fungal-type domain-containing protein |
| ATCC64974_6340 | Fungal Specific TF; Zn(2)-C6 fungal-type domain-containing protein |
| ATCC64974_39350 | Zinc finger, CCHC-type |
| **B48vsF48 up** |  |
| ATCC64974_100170 | Fungal Specific TF; Pyridine nucleotide-disulfide oxidoreductase family protein |
| ATCC64974_17730 | Fungal Specific TF; Zn(2)-C6 fungal-type domain-containing protein |
| ATCC64974_28300 | Fungal Specific TF; Zn(2)-C6 fungal-type domain-containing protein |
| ATCC64974_3940 | Fungal Specific TF; Vps16, N-terminal region family protein |
| ATCC64974_49970 | Fungal Specific TF; Zn(2)-C6 fungal-type domain-containing protein |
| ATCC64974_54630 | Fungal Specific TF; Carboxylesterase family protein |
| ATCC64974_55290 | Zinc finger, PARP-type; PARP-type domain-containing protein |
| ATCC64974_58300 | Histone-like TF; Histone H4 |
| ATCC64974_6340 | Fungal Specific TF; Zn(2)-C6 fungal-type domain-containing protein |
| ATCC64974_80200 | UAF complex subunit Rrn10 |
| ATCC64974_81740 | Homeodomain |
| ATCC64974_8490 | AT-rich interaction region; Nin one binding (NOB1) Zn-ribbon like family protein |
| ATCC64974_89080 | Fungal Specific TF; Zn(2)-C6 fungal-type domain-containing protein |
| ATCC64974_93520 | Fungal Specific TF; Fasciclin domain family protein |
| ATCC64974_95320 | Helix-turn-helix; Transcription elongation factor Spt6 |
| ATCC64974_52880 | Helix-turn-helix; Multiprotein-bridging factor 1 |
| ATCC64974_39350 | Zinc finger, CCHC-type |
| **B48vsF48 down** |  |
| ATCC64974_74060 | Helix-loop-helix; BHLH domain-containing protein |
| ATCC64974_56760 | HMG; ARID/BRIGHT DNA binding domain family protein |
| ATCC64974_93680 | Fungal Specific TF; Fungal_trans domain-containing protein |
| ATCC64974_20230 | HMG; HMG box domain-containing protein |
| ATCC64974_21020 | Histone-like +A1:B41TF; CBFD_NFYB_HMF domain-containing protein |


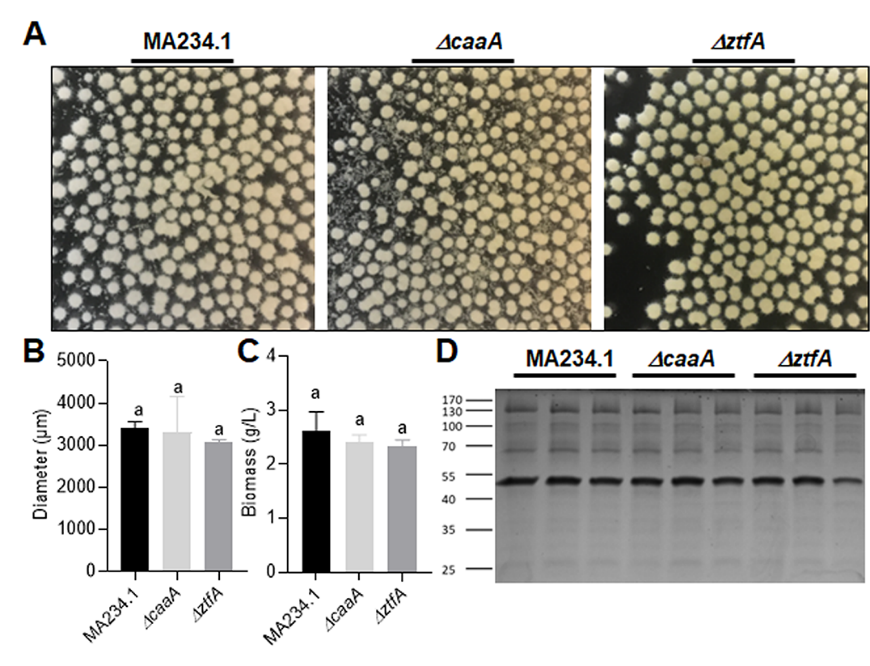


**Figure 2.** Morphology (A), diameter (B), biomass (C) and SDS PAGE of proteins in the culture medium (D) of strain MA234.1, Δ*caaA* and Δ*ztfA*. Different letters indicate statistical differences (B,C) as determined by a one-way ANOVA combined with a Games-Howell post-hoc test. Error bars indicate standard deviation.

**References**

van Leeuwe, T.M., Arentshorst, M., Ernst, T., Alazi, E., Punt, P.J., Ram, A.F.J. 2019. Efficient marker free CRISPR/Cas9 genome editing for functional analysis of gene families in filamentous fungi. *Fungal Biol Biotechnol*, **6**, 13.

Lyu, J. 2011. Functional heterogeneity of micro-colony size in *Aspergillus niger*. PhD Thesis, University of Utrecht.

Nodvig, C.S., Hoof, J.B., Kogle, M.E., Jarczynska, Z.D., Lehmbeck, J., Klitgaard, D.K., Mortensen, U.H. 2018. Efficient oligo nucleotide mediated CRISPR-Cas9 gene editing in *Aspergilli*. *Fungal Genet Biol*, **115**, 78-89.

Park, J., Park, J., Jang, S., Kim, S., Kong, S., Choi, J., Ahn, K., Kim, J., Lee, S., Kim, S., Park, B., Jung, K., Kim, S., Kang, S., Lee, Y.H. 2008. FTFD: an informatics pipeline supporting phylogenomic analysis of fungal transcription factors. *Bioinformatics*, **24**, 1024-1025.

**Supplemental Text 1C**

The secretome of *A. niger* of the different cultures was compared to the Aspertome database (<https://cb.imsc.res.in/aspertome/home>) (Vivek-Ananth et al., 2018). This database suggests that 31 proteins are secreted by the non-classical pathway (Table 1), of which 23 without a signal sequence for secretion. Of the 31 proteins, 24 were only found in the bead spore cultures, while 13 were only present in cultures with small micro-colonies (i.e. ≥ 10 h bead spore cultures).

**Table 1.** Proteins in the bead and free spore cultures that have been described to be secreted by the non-classical pathway in *Aspergilli*

| Protein ID | Gene | Annotation | Identified in |
| --- | --- | --- | --- |
| **Proteins with signal sequence** | | | |
| ATCC64974_1620 |  | Alpha galactosidase A | F6-F16, B6-B16 |
| ATCC64974_27940 |  | Eukaryotic aspartyl protease | F6-F16, B6-B16 |
| ATCC64974_67820 |  | Uncharacterized protein | F8-F16, B6-B16 |
| ATCC64974_106110 |  | SMP-30/Gluconolactonase/LRE-like region | F6-F10, F16, B6-B16 |
| ATCC64974_109710 | tilA | Multicopper oxidase | B8-B16 |
| ATCC64974_91770 |  | GH family 3 C-terminal domain | B10-B16 |
| ATCC64974_48710 |  | GH family 76 | B10-B16 |
| ATCC64974_33160 |  | Uncharacterized protein | B10-B16 |
| **Proteins without signal sequence** | | | |
| ATCC64974_79660 | apsA | ERAP1-like C-terminal domain | B6-B16 |
| ATCC64974_100780 | aldA | Aldehyde dehydrogenase family | B10-B12, B16 |
| ATCC64974_8440 | ndk1 | Nucleoside diphosphate kinase | B10-B16 |
| ATCC64974_66140 | ssb2 | Hsp70 protein | F16, B10-B16 |
| ATCC64974_87920 | alrA | Aldo/keto reductase family | B6, B10-B16 |
| ATCC64974_93080 |  | Domain of unknown function (DUF4965) | B10-B14 |
| ATCC64974_79340 |  | Enoyl-(Acyl carrier protein) reductase | B12, B16 |
| ATCC64974_12090 | leu2A | Isocitrate/isopropylmalate dehydrogenase | B10-B14 |
| ATCC64974_61280 | tpiA | Triosephosphate isomerase | B10-B14 |
| ATCC64974_54170 |  | Arylsulfotransferase (ASST) family protein | B10-B16 |
| ATCC64974_27170 |  | Thioredoxin | B6-B16 |
| ATCC64974_53350 |  | GH family 38 N-terminal domain | B6-B16 |
| ATCC64974_10730 |  | Prolyl oligopeptidase family | B10-B16 |
| ATCC64974_76670 |  | Peptidase family M49 | B8-B16 |
| ATCC64974_7620 |  | ERAP1-like C-terminal domain | B8-B16 |
| ATCC64974_105620 |  | Aminotransferase class-V | B6, B10-B16 |
| ATCC64974_84190 |  | TAP-like protein | B10-B16 |
| ATCC64974_23160 |  | Eukaryotic aspartyl protease | F6, F10-F16, B6-B16 |
| ATCC64974_109650 |  | Serine aminopeptidase, S33 | F8-F12, F16, B6-B16 |
| ATCC64974_3650 |  | GH family 92 | B8-B16 |
| ATCC64974_69070 | ssc1 | Hsp70 protein | B8-B16 |
| ATCC64974_68870 | pgi1 | Phosphoglucose isomerase | B8-B16 |
| ATCC64974_85000 | glr1 | Pyridine nucleotide-disulphide oxidoreductase | B10-B16 |

**References**

Vivek-Ananth, R.P., Mohanraj, K., Vandanashree, M., Jhingran, A., Craig, J.P., Samal, A. 2018. Comparative systems analysis of the secretome of the opportunistic pathogen *Aspergillus fumigatus* and other *Aspergillus* species. *Sci Rep*, **8**, 6617.
