# Supplementary material for: Heterogeneity in Spore Aggregation and Germination Results in Different Sized, Cooperative Microcolonies in an Aspergillus niger Culture
Source: mBio. 2023 Jan 11;14(1):e00870-22. doi: 10.1128/mbio.00870-22 (PMC9973262; doi:10.1128/mbio.00870-22)
Supplement: TABLE S1 [file mbio.00870-22-s0001.docx]

**Supplemental Table 1**. Primers used in this study.

| Primer | Sequence |
| --- | --- |
| actFwd | GTTGCTGCTCTCGTCATT |
| actRev | AACCGGCCTTGCACATA |
| 14700Fwd | AGAAGATCCTAAGCAAGCGA |
| 14700Rev | ATTGATGGAAGCCGAAAGTC |
| 21820Fwd | GAGCAAGTCATGTTCCAACC |
| 21820Rev | GAGGTACGAGAAGAGACGG |
| 48070Fwd | CCGTTCCCTCTACCTTCGT |
| 48070Rev | ATCGTGTTCGCCTGACTC |
| 58540Fwd | GTACCACCGTCATCTTCGAG |
| 58540Rev | CGCAGTTGATCTTGGCAC |
| 96170Fwd | CCAAGCTGATCCTCTCCTCC |
| 96170Rev | GCACCGTACCAGTAGACCT |
| 67930Fwd | CGATACCAACAACGAGTACC |
| 67930Rev | TAGTCCAGTCCCAGGCA |
